# Analysis of ubiquitin recognition by the HECT ligase E6AP provides insight into its linkage specificity

**DOI:** 10.1074/jbc.RA118.007014

**Published:** 2019-02-08

**Authors:** Lena K. Ries, Bodo Sander, Kirandeep K. Deol, Marie-Annick Letzelter, Eric Robert Strieter, Sonja Lorenz

**Affiliations:** From the ‡ Rudolf Virchow Center for Experimental Biomedicine, University of Würzburg, 97080 Würzburg, Germany and; the Departments of § Chemistry and; ¶ Biochemistry and Molecular Biology, University of Massachusetts at Amherst, Amherst, Massachusetts 01003

**Keywords:** ubiquitin, ubiquitin ligase, ubiquitylation (ubiquitination), post-translational modification, enzyme mechanism

## Abstract

Deregulation of the HECT-type ubiquitin ligase E6AP (UBE3A) is implicated in human papilloma virus-induced cervical tumorigenesis and several neurodevelopmental disorders. Yet the structural underpinnings of activity and specificity in this crucial ligase are incompletely understood. Here, we unravel the determinants of ubiquitin recognition by the catalytic domain of E6AP and assign them to particular steps in the catalytic cycle. We identify a functionally critical interface that is specifically required during the initial formation of a thioester-linked intermediate between the C terminus of ubiquitin and the ligase-active site. This interface resembles the one utilized by NEDD4-type enzymes, indicating that it is widely conserved across HECT ligases, independent of their linkage specificities. Moreover, we uncover surface regions in ubiquitin and E6AP, both in the N- and C-terminal portions of the catalytic domain, that are important for the subsequent reaction step of isopeptide bond formation between two ubiquitin molecules. We decipher key elements of linkage specificity, including the C-terminal tail of E6AP and a hydrophilic surface region of ubiquitin in proximity to the acceptor site Lys-48. Intriguingly, mutation of Glu-51, a single residue within this region, permits formation of alternative chain types, thus pointing to a key role of ubiquitin in conferring linkage specificity to E6AP. We speculate that substrate-assisted catalysis, as described previously for certain RING-associated ubiquitin–conjugating enzymes, constitutes a common principle during linkage-specific ubiquitin chain assembly by diverse classes of ubiquitination enzymes, including HECT ligases.

## Introduction

Ubiquitination controls the lifetimes, conformational dynamics, as well as the localization and interaction patterns of eukaryotic proteins, thereby directing cellular functions at all levels. The molecular basis for this regulatory potential lies in the finely-tuned interplay of ubiquitinating and de-ubiquitinating activities, which modify substrate proteins with ubiquitin in reversible and highly-specific manners. The structural mechanisms encoding specificity in ubiquitination reactions, however, are incompletely understood.

Ubiquitination is driven by a cascade of ubiquitin-activating enzymes (E1),^5^ ubiquitin-conjugating enzymes (E2), and ubiquitin ligases (E3). The plethora of ligases—over 600 have been estimated in the human proteome (1)—have pivotal roles in conferring specificity in ubiquitination, because they select substrates and modification sites and often determine the types of modifications that are generated. Ubiquitin ligases typically catalyze the formation of an isopeptide or peptide linkage between the C terminus of ubiquitin and a primary amino group of a substrate. If ubiquitin itself acts as a substrate, chains linked through either of eight primary amino groups (seven lysine residues and the N terminus) can be formed. Notably, individual linkage types give rise to particular chain conformations, which, in turn, trigger distinct physiological outcomes.

Based on their architectures, ubiquitin ligases follow different mechanisms: RING (Really Interesting New Gene)- and U-box ligases catalyze the direct transfer of ubiquitin from a thioester-linked complex with an E2 to a substrate. In contrast, HECT (Homologous to E6AP C terminus) and RBR (RING-between-RING) ligases act through a catalytic cysteine and form a thioester-linked intermediate with ubiquitin before modifying the substrate (2, 3).

The structural determinants of linkage-specific ubiquitin chain formation were delineated for several E2/RING systems and the RBR-type ligase HOIP (4–8). In contrast, it has remained elusive at a structural level how HECT ligases achieve linkage specificity. Yet it is evident from the distinct linkage preferences of individual members that different modes of ubiquitin recognition exist within the HECT ligase family. For example, UBE3A (hereafter referred to as “E6AP”) assembles Lys-48–linked chains (9–11), thereby targeting substrates for proteasomal degradation; UBE3C and AREL1 synthesize mixed-linkage chains, which contain mostly Lys-48/29 and Lys-33/11 linkages, respectively (12–15); and NEDD4-type enzymes as well as HECTD1 are Lys-63 linkage–specific or display context-dependent preferences (10, 11, 16–26); variable linkage selectivity has also been reported for HUWE1 (27–29).

During ubiquitin chain formation, the ubiquitin molecule that is thioester-linked to the catalytic cysteine of the ligase is referred to as the “donor”; the ubiquitin molecule that presents a primary amino group for the nucleophilic attack on the C terminus of the donor is known as the “acceptor.” The orientation of the acceptor toward the donor determines which primary amino group is modified and thus holds the key to specificity in linkage formation and associated signaling responses. Yet, how this mechanism is structurally implemented in HECT ligases is not entirely clear.

HECT ligases share a C-terminal catalytic “HECT” domain (∼45 kDa), which is composed of two lobes (30): the N-terminal lobe (“N-lobe”) mediates E2 binding (30–33) and, at least in some cases, interactions with a regulatory ubiquitin molecule (18, 26, 34–39); the C-terminal lobe (“C-lobe”) contains the catalytic cysteine and interacts with both the donor and the acceptor ubiquitin (11, 18, 30–32, 40, 41). Although structural studies have shown that several NEDD4-type ligases and HUWE1, whose HECT domain is closely related to the NEDD4 subfamily, use a common binding mode for the donor ubiquitin (18, 31, 40, 41), it is unknown whether this mode is conserved across evolutionarily more distant HECT ligases. With regard to the recognition of the acceptor ubiquitin, there is evidence suggesting that the determinants of linkage specificity reside in the C-lobe (11, 18), but the molecular details are poorly understood. A major challenge in unraveling how the C-lobes of HECT ligases recognize their ubiquitin substrates lies in the fact that functionally critical interactions are rather weak (high micromolar to millimolar *K_D_* range).

In this study, we combine NMR spectroscopy with mutational analyses and absolute quantification (AQUA) MS to decipher the mechanism of ubiquitin recognition by E6AP. This ligase regulates key cellular processes, including translation, DNA replication, and intracellular trafficking (42), and is critical in diverse human pathogeneses. For one, E6AP is hijacked by the E6 protein from high-risk human papilloma viruses to promote the proteasomal degradation of the tumor suppressor p53, thereby driving cervical cancer (43–45). Moreover, genetic amplification or mutational up-regulation of E6AP is associated with autism-spectrum disorders, and deletion or down-regulation of this ligase in the brain results in a neurodevelopmental disease known as Angelman's syndrome (45, 46). Although E6AP was the first ubiquitin ligase shown to function through a thioester intermediate (2) and its HECT domain to be structurally characterized (30), the structural basis of catalysis in E6AP is still incompletely understood; this has precluded rational approaches to target this ligase therapeutically (47).

Here, we demonstrate that the HECT domain of E6AP relies on canonical, “NEDD4-type” contacts with the donor ubiquitin during thioester formation. We also identify surface patches on ubiquitin and E6AP critical for the subsequent step of isopeptide bond formation, and we uncover determinants of the Lys-48 linkage specificity of E6AP. Intriguingly, these determinants reside in both the ligase and ubiquitin itself, which underscores the widespread role of substrate-assisted catalysis in ubiquitination reactions. Finally, we reveal that the N-lobe of E6AP interacts with ubiquitin and that the exosite region is required for isopeptide bond formation and influences ubiquitin binding, in a similar yet not identical manner as characterized for NEDD4 ligases.

## Results

### E6AP C-lobe recognizes ubiquitin in trans

**Figure 1.**
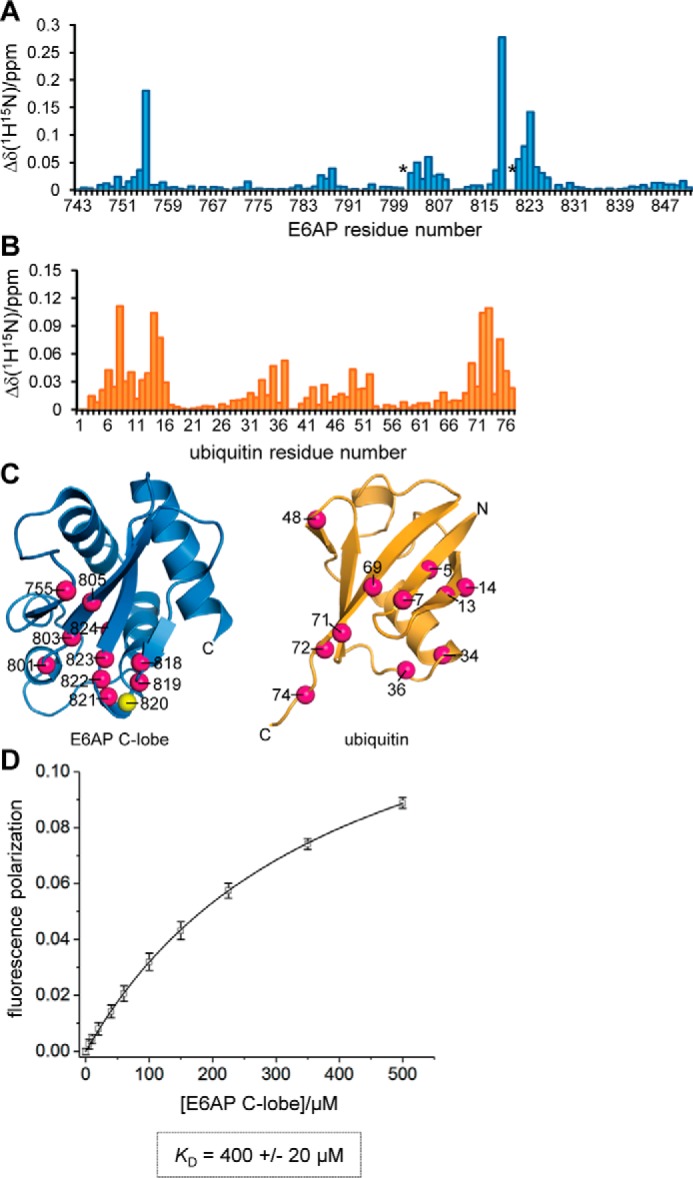
**C-lobe of E6AP interacts with ubiquitin in *trans*.** *A,* weighted and combined chemical shift perturbations, Δδ(^1^H^15^N), of E6AP C-lobe resonances induced by a 12.5-fold molar excess of ubiquitin, plotted over the E6AP residue number. Resonances that undergo line broadening (Lys-801 and Thr-819) are marked by an *asterisk. B,* weighted, combined chemical shift perturbations of ubiquitin resonances induced by a 12.5-fold molar excess of the E6AP C-lobe plotted over the ubiquitin residue number. *C,* structures of the E6AP C-lobe (extracted from PDB code 1C4Z (30)) and ubiquitin (PDB code 1UBQ (94)) are shown in *ribbon* representation. The nitrogen atoms of backbone amide groups whose resonances display binding-induced shift perturbations, Δδ(^1^H^15^N) > 0.04, or undergo line broadening (Lys-801 and Thr-819 of E6AP) are highlighted as *balls* (*magenta*). The side chain of the catalytic cysteine, Cys-820, is also displayed. *D,* determination of an apparent dissociation constant, *K_D_*, for the C-lobe–ubiquitin interaction based on FP measurements. The mean FP signal and standard deviations from three independent experiments using a fluorophore-labeled ubiquitin variant were plotted as a function of the C-lobe concentration and fitted to a single-site binding model (*line*).

During the catalytic cycle of HECT ligases, the C-lobe recognizes both the donor and acceptor ubiquitin in *trans* (11, 31). However, because of their transient nature, these interactions have escaped detection in pulldown experiments (11, 31, 37). We thus employed NMR spectroscopy to monitor weak interactions between the C-lobe of E6AP and ubiquitin. Indeed, we observed binding-induced chemical shift perturbations in ^1^H-^15^N HSQC spectra of the ^15^N-enriched C-lobe upon addition of ubiquitin and vice versa, indicating a specific interaction (Fig. 1, *A* and *B*). The affected resonances map to mostly continuous surface areas on the two binding partners. On ubiquitin, this area includes several hydrophobic residues (Val-5, Ile-13, Ile-36, Leu-69, and Leu-71) as well as residues in the flexible C-terminal tail (Arg-72 and Arg-74) (Fig. 1*C*). Perturbed resonances of E6AP map primarily to residues in the active-site region surrounding the catalytic cysteine (Cys-820), such as His-818, Thr-819, Phe-821, Asn-822, Val-823, and Leu-824, with additional perturbations seen for Gly-755, Lys-801, Ile-803, and Ala-805 (Fig. 1*C*).

Based on fluorescence polarization (FP) measurements with fluorophore-labeled ubiquitin, we determined an apparent dissociation constant, *K_D_*, of 400 ± 20 μm for the C-lobe–ubiquitin interaction (Fig. 1*D*). This rather weak affinity explains why the interaction had not been directly detected in previous assays (11). Moreover, it is reminiscent of the interactions of isolated E2s with donor and acceptor ubiquitin, respectively, that typically fall into a near-millimolar *K_D_* range, despite being functionally critical (48).

### E6AP relies on NEDD4-type contacts with the donor ubiquitin during thioester formation

**Figure 2.**
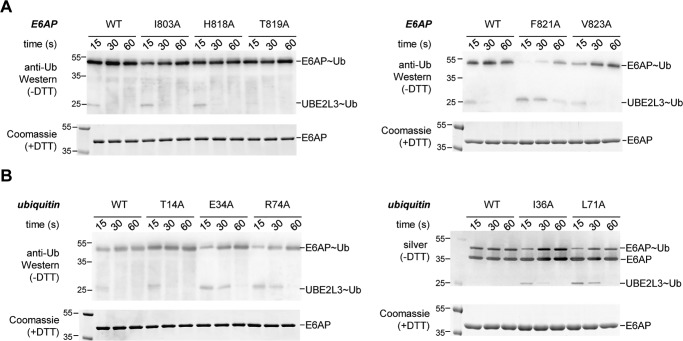
**Most of the residues identified by NMR are important for thioester transfer of ubiquitin from UBE2L3 to E6AP.** *A,* thioester transfer of ubiquitin from the E2 (UBE2L3) to the E6AP HECT domain, followed in single-turnover, pulse-chase assays at three time points, as indicated, and monitored by nonreducing SDS-PAGE and anti-ubiquitin Western blotting. The thioester-linked HECT domain–ubiquitin conjugate (*E6AP*∼*Ub*) and, in some cases, the thioester-linked E2-ubiquitin precursor (*UBE2L3*∼*Ub*) are visible. The input amount of HECT domain (*E6AP*) is monitored by reducing SDS-PAGE and Coomassie staining. Note that no auto-ubiquitination of the HECT domain occurs within the tested time range. *B,* analogous assays as in *A*, monitoring the effect of mutations in ubiquitin on thioester transfer to the E6AP HECT domain. For the I36A and L71A variants, silver staining (*right*) was used in lieu of anti-ubiquitin Western blotting (*left*), because these variants are not detected well by the antibody (P4D1) used here.

To interrogate the functional significance of the identified E6AP–ubiquitin interaction, we introduced individual alanine mutations at those positions that displayed the largest binding-induced chemical shift perturbations. Those include Ile-803, His-818, Thr-819, Phe-821, and Val-823 of E6AP (Gly-755 was not mutated for structural reasons nor was Lys-801, Asn-822, and Leu-824, due to their side chains being buried) and Thr-14, Glu-34, Ile-36, Leu-71, and Arg-74 of ubiquitin. The purified HECT domain variants were tested for their ability to receive the donor ubiquitin from the cognate E2 (UBE2L3) in thioester transfer assays (Fig. 2, *A* and *B*). Based on time-course experiments, monitoring both the formation of the thioester-linked HECT domain–ubiquitin conjugate (“E6AP∼Ub”) and the concomitant loss of the E2–ubiquitin conjugate (“UBE2L3∼Ub”), we found that the mutations I803A, H818A, and T819A in E6AP do not impair thioester transfer (Fig. 2*A*, *left*). In contrast, the F821A substitution adjacent to the catalytic cysteine causes a strong defect, as seen from a delay in the formation of the E6AP∼Ub product and the prolonged presence of the UBE2L3∼Ub precursor. The V823A variant is also compromised in thioester transfer, albeit to a smaller degree than F821A (Fig. 2*A*, *right*).

With the exception of T14A, all tested mutations in ubiquitin markedly suppress thioester transfer to the E6AP HECT domain (Fig. 2*B*). Notably, these defects are specific to the transfer of ubiquitin from the E2 to the E3, because the preceding reaction step, as monitored by the E1-mediated formation of UBE2L3∼Ub, is unaffected by the mutations (Fig. S1), in line with previous analyses (49).

**Figure 3.**
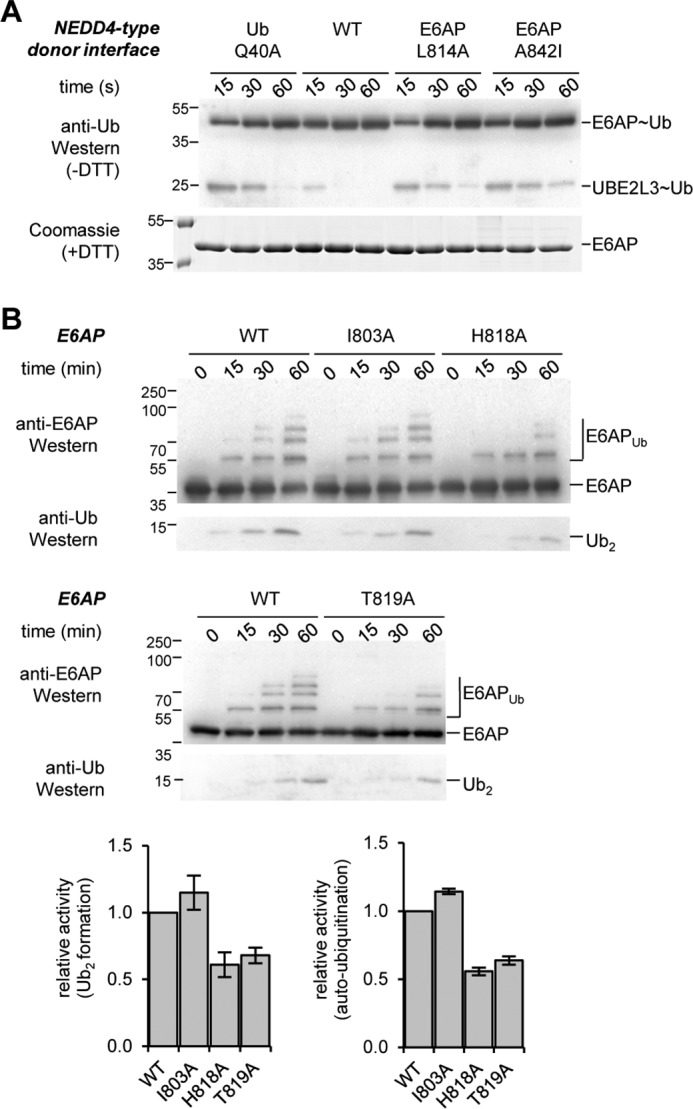
**Thioester transfer of ubiquitin requires similar residues in E6AP and NEDD4-type ligases; alternative residues are involved in isopeptide bond formation.** *A,* thioester transfer of ubiquitin from the E2 (UBE2L3) to the E6AP HECT domain interrogating additional variants of ubiquitin (Q40A) and E6AP (L814A and A842I). The mutation sites are homologous to critical residues in the donor interface of NEDD4-type ligases (18, 31, 40, 41). *B,* isopeptide bond formation assays comparing the activities of the E6AP HECT domain WT and those variants tested in Fig. 2*A* that do not display defects in thioester formation. Activities are monitored at three time points, as indicated, by reducing SDS-PAGE and Western blotting against E6AP (HECT domain auto-ubiquitination marked as *E6AP_Ub_*) and ubiquitin (di-ubiquitin reaction product marked as *Ub_2_*), respectively. Time point 0 denotes samples before ATP addition. The amounts of Ub_2_ and E6AP_Ub_ were quantified and normalized to the input amount of E6AP. Quantifications are based on three independent experiments; the means and standard deviations were plotted for the 60-min time point.

Interestingly, the residues of ubiquitin required for thioester transfer to E6AP coincide with a surface of the donor ubiquitin that engages the C-lobe of NEDD4-type ligases (18, 31, 40, 41). We thus speculated that E6AP associates with the donor ubiquitin in a similar manner. To test this hypothesis, we introduced mutations known to interfere with donor recognition by NEDD4-type ligases at the homologous positions in E6AP: Leu-814 (homologous to Leu-861 and Leu-916 of NEDD4 and NEDD4L, respectively) (31, 40, 41); and Ala-842 (homologous to Ala-889 and Ala-944 of NEDD4 and NEDD4L, respectively) (18, 31). Another mutation reported to inhibit thioester transfer to NEDD4L (F881A, homologous to F785A in E6AP) (31) was also introduced into E6AP, but it could not be studied further, because it reduced the stability of the HECT domain (data not shown). In line with the notion that donor ubiquitin recognition by E6AP resembles that of NEDD4-type ligases during thioester transfer, we found that the L814A and A842I HECT domain variants are impaired in thioester formation with ubiquitin compared with the wildtype (WT) (Fig. 3*A*). Consistently, thioester formation is also delayed upon mutation of Gln-40 in ubiquitin (Q40A), which makes key contacts at the NEDD4-type donor ubiquitin interface (Fig. 3*A*). However, Gln-40 is not required for thioester transfer of ubiquitin from the E1 to the E2 (Fig. S1).

We also studied the functional significance of those residues of E6AP that displayed significant ubiquitin-induced chemical shift perturbations in our NMR experiments but are not required for thioester transfer of ubiquitin (Ile-803, His-818, and Thr-819; see Fig. 2*A*). To this end, we monitored the second step in the catalytic cycle of HECT ligases, isopeptide bond formation, by virtue of auto-ubiquitination (“E6AP_Ub_”) and di-ubiquitin formation (“Ub_2_”) (Fig. 3*B*). These studies show that the mutation of residues in close proximity to the catalytic cysteine (H818A and T819A) causes defects in isopeptide bond formation activity compared with the WT. In contrast, Ile-803, which borders a β-strand flanking the active-site region, has similar activity to the WT.

These observations are consistent with the notion that the ubiquitin-induced chemical shift perturbations of E6AP backbone amide resonances report on both direct contacts with ubiquitin, which occur predominantly at the active-site region, and conformational changes propagated to the nearby β-sheet. Similarly, solution studies of donor ubiquitin conjugated to the C-lobe of SMURF2 or of HUWE1 revealed conformational changes in the C-lobes remote from the crystallographic donor interface (41). However, it cannot be ruled out that our NMR data reflect more than one binding mode between the C-lobe of E6AP and ubiquitin in *trans*.

**Figure 4.**
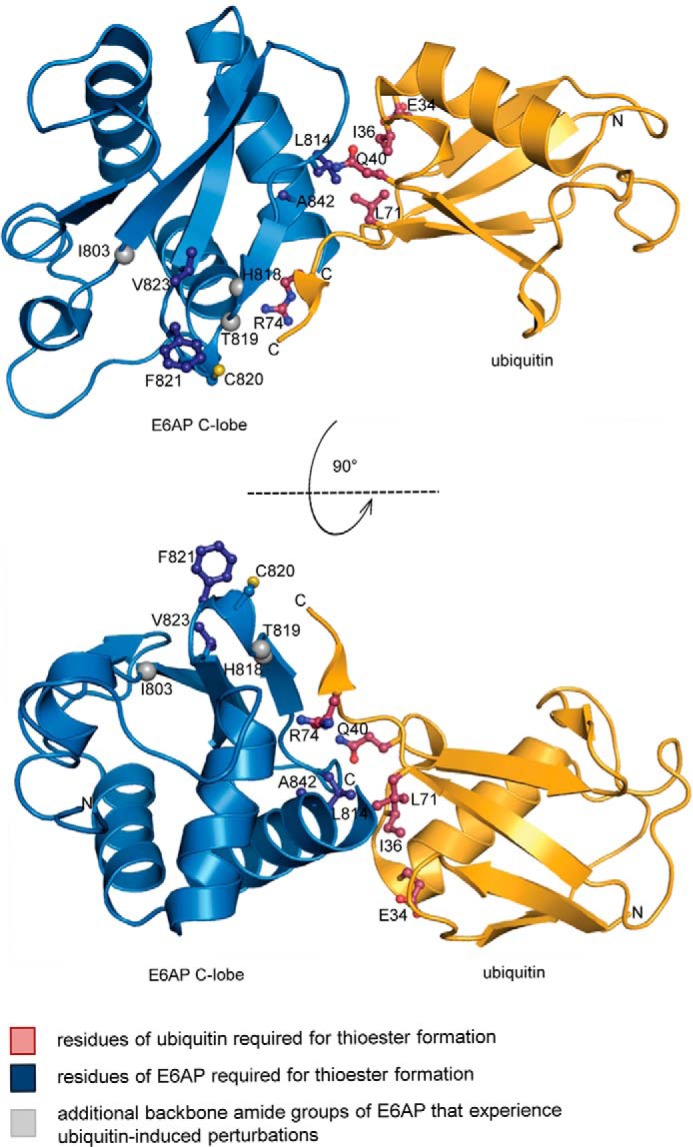
**Model of a NEDD4-type complex between the C-lobe of E6AP and ubiquitin, as required for thioester formation.** Ubiquitin was modeled by structural superposition of the C-lobe of the ubiquitin-bound HECT domain of NEDD4 (PDB code 4BBN, chain A (18)) with the C-lobe of the HECT domain of E6AP (chain A, extracted from PDB code 1C4Z (30)), using the PyMOL Molecular Graphics System, Version 2.0, Schrödinger, LLC. Ubiquitin and the E6AP C-lobe are displayed in *ribbon* representation; the side chains of residues relevant for thioester formation are displayed as *balls and sticks*. The side chain of the catalytic cysteine, Cys-820, is also displayed. The backbone nitrogen atoms of additional residues that experience perturbations upon ubiquitin addition are shown as *spheres*.

Taken together, we conclude that the C-lobe of E6AP relies on similar contacts with ubiquitin as NEDD4-type ligases for thioester formation with ubiquitin (18, 31, 40, 41). A structural model, shown in Fig. 4, features the functionally validated residues, as well as those backbone amide groups that either experience propagated structural perturbations or are involved in alternative, yet uncharacterized interaction modes.

### C-terminal tail of E6AP interacts with the donor ubiquitin upon thioester formation and directs linkage specificity

**Figure 5.**
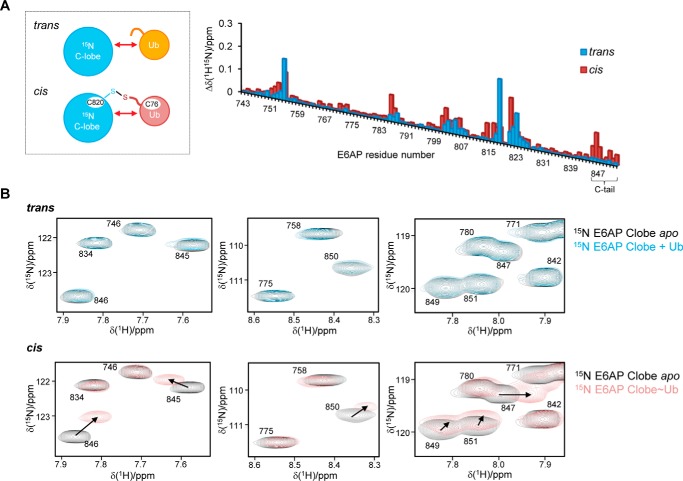
**C-terminal tail of E6AP interacts with the donor ubiquitin upon thioester formation.** *A,* schematic of the protein samples used to monitor the interactions of the ^15^N-enriched C-lobe of E6AP and ubiquitin in *trans* or in the context of a disulfide-linked complex containing ubiquitin G76C (*left*). Weighted and combined chemical shift perturbations, Δδ(^1^H^15^N) of C-lobe resonances, were induced by ubiquitin in *trans* (12.5-fold molar excess) or *cis* plotted over the E6AP residue number (*right*). The functionally important C-tail region is marked. *B,* sections of the ^1^H-^15^N BEST-TROSY spectra (as analyzed in *A*) featuring resonances that originate from the C-tail. Each section shows a superposition of spectra in the presence (*blue* and *rose,* respectively) and absence (*black*) of ubiquitin.

We next set out to analyze the interaction of the E6AP C-lobe with the donor ubiquitin in the context of a covalently linked intermediate, as formed during the reaction cycle. We thus prepared a stable mimic of the conjugate by replacing the native thioester with a disulfide bond (Fig. 5*A*, *left*) and compared the pattern of chemical shift perturbations of C-lobe resonances induced by ubiquitin in *cis* with those seen in *trans* (see Fig. 1*A*). In quantitative terms, this analysis yields overall rather similar chemical shift perturbations (Fig. 5*A*, *right*). This is consistent with the FP-derived affinity for the interaction (see Fig. 1*D*). At the given protein concentrations (200 μm C-lobe; 12.5-fold molar access of ubiquitin) and a *K_D_* value of 400 μm, a complex saturation of ∼85% is expected. However, the generally small amplitudes and limited number of chemical shift perturbations of E6AP resonances indicate that the donor ubiquitin retains considerable flexibility upon conjugation to the C-lobe.

Addition of high concentrations (up to 3 mm) of unlabeled ubiquitin to the C-lobe–donor conjugate slightly enhances the chemical shift perturbations of C-lobe resonances (Fig. S2). This likely reflects an increased saturation of the C-lobe–donor complex in the presence of added ubiquitin concentrations that approach the local concentrations of the covalently linked donor at the C-lobe. A second ubiquitin-binding site on the C-lobe is not detected, indicating that interactions with the acceptor ubiquitin are extremely weak under these conditions and require additional components to be stabilized. As noted above, however, it is impossible to decide unequivocally whether the observed chemical shift perturbations result from a single or several dynamic binding modes.

As seen in *trans*, major chemical shift perturbations in *cis* affect residues in the active-site region (residues 818–823) and at the rim of the flanking β-sheet (residues 755 and 802–807) (Fig. 5*A*, *right*). Several resonances undergo pronounced chemical shift perturbations in *cis*: those correspond to Thr-786, Thr-787, Lys-801, Met-802, and Phe-821, all of which are located in structural elements near the catalytic cysteine where the C-terminal tail of ubiquitin is attached; and Leu-814 and Thr-816, which fall into the canonical donor interface, in line with our model of thioester formation (Fig. 4). Notably, specific chemical shift perturbations in *cis* are also observed in the C-terminal region of E6AP (“C-tail”; residues 845–852) (Fig. 5, *A* and *B*), indicating that the C-tail contributes to interactions with the donor, once a covalent linkage between the C-lobe and ubiquitin has been established.

**Figure 6.**
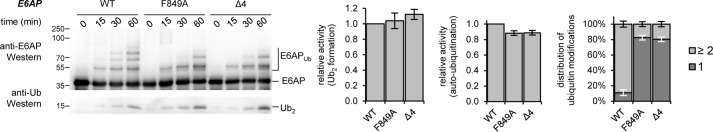
**C-terminal tail of E6AP is essential for ubiquitin chain elongation.** Isopeptide bond formation assays compare the activities of the E6AP HECT domain WT and C-terminal tail variants. Activities are monitored at three time points, as indicated, by reducing SDS-PAGE and Western blotting against E6AP (HECT domain auto-ubiquitination marked as *E6AP_Ub_*) and ubiquitin (di-ubiquitin reaction product marked as *Ub_2_*), respectively. Time point 0 denotes samples before ATP addition. The amounts of Ub_2_ and unmodified E6AP were quantified and normalized to the input amount of E6AP. In addition, the contributions of mono-ubiquitination (1 ubiquitin moiety) and chain formation/multimono-ubiquitination (≥2 ubiquitin moieties) were quantified for each variant individually (with the total amount set to 1). Quantifications are based on three independent experiments; the means and standard deviations were plotted for the 60-min time point.

To dissect the function of the C-tail of E6AP, we generated mutated variants of the HECT domain in which a conserved phenylalanine, Phe-849, four residues from the C terminus is replaced by alanine (F849A), or the four C-terminal residues are deleted (“Δ4”). In line with previous mutational analyses, both variants promote thioester formation with the donor ubiquitin to a similar degree as the WT (Fig. S3*A*) (50, 51). Moreover, the integrity of the C-tail does not affect the reactivity of the catalytic cysteine in the context of the isolated C-lobe. We determined identical p*K_a_* values of 8.7 ± 0.1 for the thiol group of Cys-820 in the WT and Δ4 variant, resembling the typical value for an unperturbed cysteine (Fig. S3*B*) (52). In regard to isopeptide bond formation, both HECT domain variants form Ub_2_ with similar efficiencies as the WT (Fig. 6). However, the variants show slightly reduced activity in overall auto-ubiquitination, as quantified by the turnover of unmodified E6AP, and a pronounced defect in chain elongation during auto-ubiquitination (Fig. 6).

**Figure 7.**
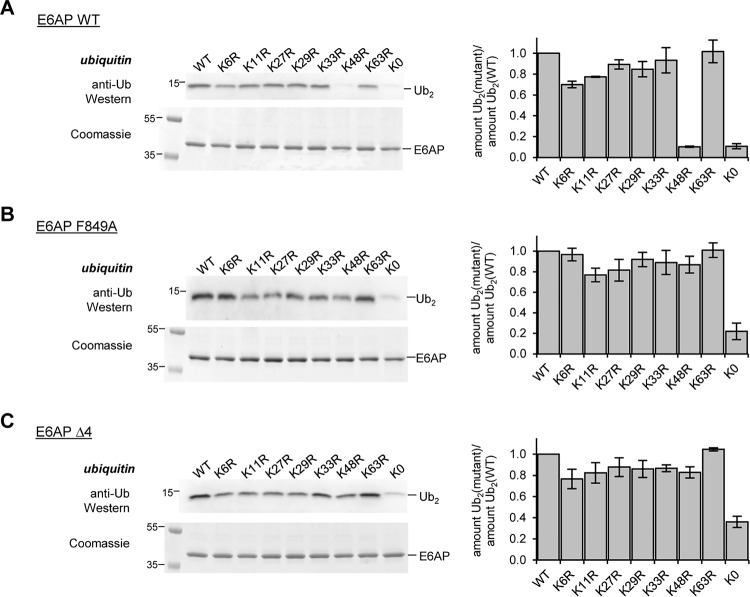
**C-terminal tail of E6AP directs Lys-48 specificity of ubiquitin linkage formation.** *A–C,* assays monitoring the activities of the E6AP HECT domain WT and C-terminal tail variants toward ubiquitin WT, single Lys-to-Arg variants, or a lysine-free (*K0*) variant, respectively, by reducing SDS-PAGE and Western blotting against ubiquitin (di-ubiquitin reaction product marked as *Ub_2_*). Assays were conducted for 15 min; the input amount of HECT domain (*E6AP*) in the absence of ATP is monitored by reducing SDS-PAGE and Coomassie staining (*left*). The amount of Ub_2_ was quantified, and the ratio of the amounts of mutated Ub_2_ to WT Ub_2_ was plotted. Quantifications are based on three independent experiments; the means and standard deviations were plotted for the 60-min time point (*right*).

These observations prompted us to examine whether the C-tail influences the recognition of the acceptor ubiquitin and thus the linkage specificity of E6AP. We compared the activities of the HECT domain variants (WT, F849A, and Δ4) toward WT ubiquitin, individual Lys-to-Arg variants (K6R, K11R, K27R, K29R, K33R, K48R, and K63R), and a lysine-free variant (“K0”; all lysine residues replaced by arginine), respectively (Fig. 7, *A–C*). These studies reveal that the C-tail variants promote Ub_2_ formation with each of the single Lys-to-Arg variants of ubiquitin, including K48R. In contrast, the WT HECT domain is highly selective for the formation of Lys-48 linkages, in line with previous studies (9–11). Notably, K0 ubiquitin is a poor substrate for all three E6AP variants, indicating that linkage formation through the N-terminal amino group of ubiquitin is generally disfavored, regardless of the integrity of the C-tail (Fig. 7, *A–C*).

**Figure 8.**
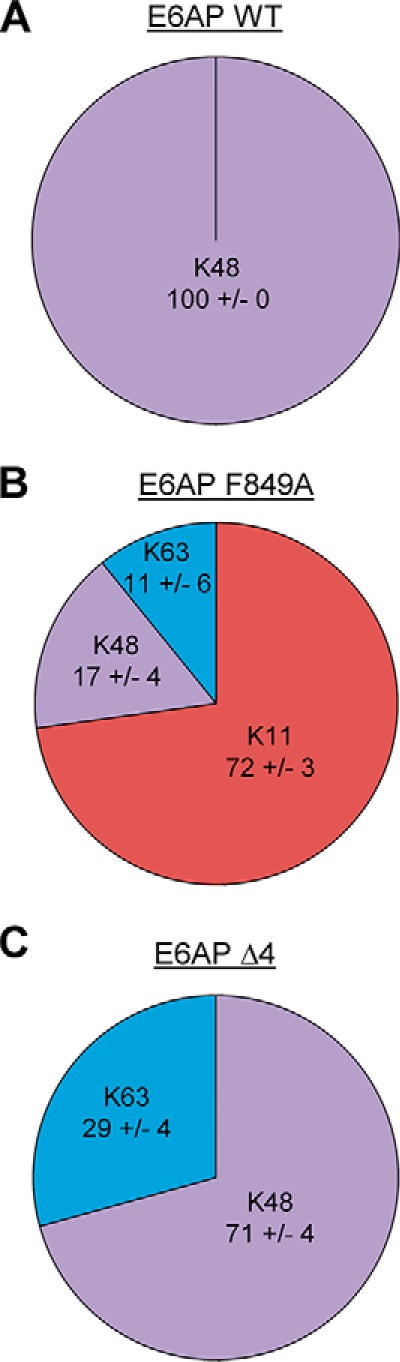
**Quantification of E6AP-catalyzed ubiquitin linkage types upon mutation of the C-terminal tail of the ligase.** AQUA mass spectrometric analysis of Ub_2_ species formed by the E6AP HECT domain WT (*A*), F849A (*B*), and Δ4 (*C*) variants. Results were normalized to the total amount of ubiquitin for each linkage type detected; the means and standard deviations from three replicates are shown (in %). The corresponding data are provided in Fig. S4.

To corroborate these findings, we analyzed the linkage composition of Ub_2_ species assembled by the WT, F849A, and Δ4 HECT domain variants by AQUA MS (absolute quantification MS) (Fig. 8 and Fig. S4, *A–C*). These analyses confirm that the WT HECT domain is Lys-48–specific. Alteration of the C-tail, however, results in a loss of specificity, as evidenced by the formation of Lys-11 and Lys-63 linkages. Notably, these data illustrate also that studies on Lys-to-Arg variants of ubiquitin do not exactly mirror the results of Ub-AQUA MS analyses (performed in the context of WT ubiquitin), consistent with previous reports, cautioning direct correlation of these two types of measurements (53).

Taken together, we conclude that the C-tail of E6AP, mediated by Phe-849, contacts the donor ubiquitin in the covalently linked intermediate and contributes to Lys-48 linkage specificity of chain formation by restricting interactions with the acceptor ubiquitin.

### Hydrophilic surface near Lys-48 is critical for acceptor ubiquitin function with E6AP

**Figure 9.**
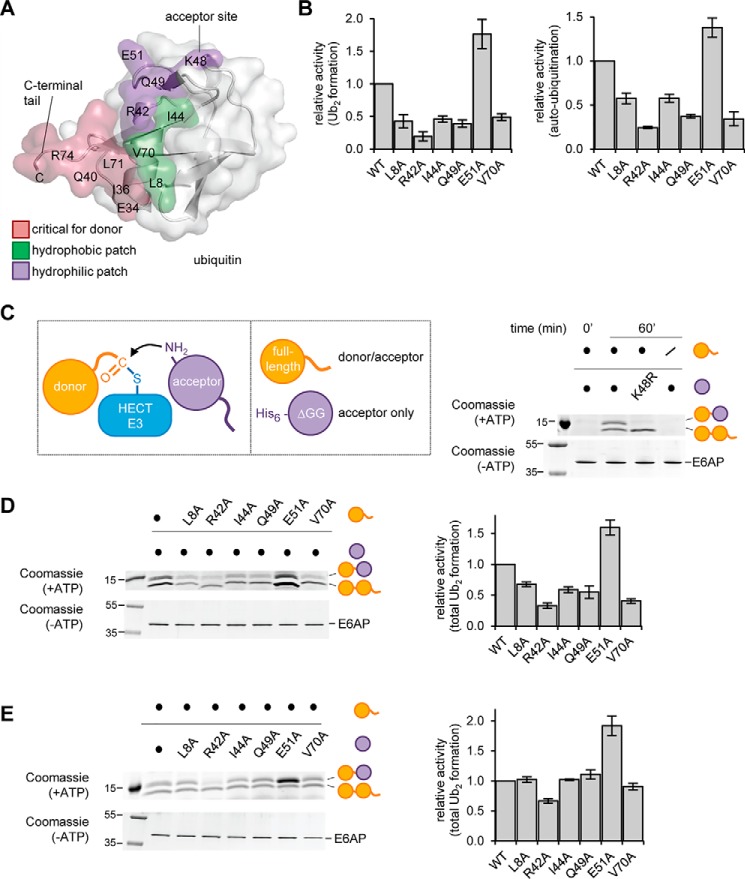
**Distinct surface regions are required by the acceptor and donor ubiquitin during E6AP-catalyzed ubiquitin linkage formation.** *A*, structure of ubiquitin (PDB code 1UBQ (94)) is shown in *ribbon* and *surface* representation. The area of the donor ubiquitin, including the C-terminal tail, that contacts the HECT C-lobe in the canonical mode is colored *rose*; hydrophobic residues important for E6AP activity are in *green* (49); and residues in proximity of Lys-48, as studied in *B–D*, are in *purple. B,* quantification of the isopeptide bond formation activity of the E6AP HECT domain toward ubiquitin variants, based on three independent experiments (for experimental data, see Fig. S5). For free chain formation (*left*) and auto-ubiquitination (*right*), the amount of reaction product (Ub_2_ and E6AP_Ub_, respectively) at 60 min was normalized to that of input E6AP. *C,* schematic of HECT ligase-mediated linkage formation between an enzyme-linked donor and an acceptor ubiquitin (*left*), and the two ubiquitin substrates employed in the Δ*GG* assay: WT ubiquitin and His_6_-tagged, truncated ubiquitin (residues 1–74; Ub^ΔGG^) (*middle*); validation of the assay using the E6AP HECT domain and equimolar mixtures of the indicated ubiquitin substrates. The Ub_2_ reaction products were analyzed by reducing SDS-PAGE and Coomassie staining. The input amount of E6AP in the absence of ATP serves as a control (*right*). *D* and *E,* ΔGG assay with ubiquitin variants, performed and analyzed as in *C*. The amount of both Ub_2_ products combined were quantified and normalized to the amount of input E6AP, and the means and standard deviations from three independent experiments were plotted (*right*).

To further decipher the requirements of acceptor ubiquitin recognition during E6AP-mediated ubiquitin linkage formation, we focused on an array of hydrophobic residues of ubiquitin (Leu-8, Ile-44, and Val-70), often referred to as the “hydrophobic patch” that is critical for E6AP activity (49). Because these residues do not fall into the canonical donor–C-lobe interface relevant during thioester formation (Fig. 9*A*), we hypothesized that they may be required for alternative interactions of the donor or the acceptor ubiquitin. In addition, we decided to interrogate the functional significance of a series of hydrophilic residues of ubiquitin in proximity to the Lys-48–acceptor site, including Arg-42, Gln-49, and Glu-51 (Fig. 9*A*).

We generated ubiquitin variants with individual alanine substitutions at the selected sites and monitored isopeptide bond formation, as catalyzed by the E6AP HECT domain. These studies confirm that residues in the hydrophobic patch of ubiquitin, as well as Arg-42 and Gln-49, are important for E6AP activity (Fig. 9*B* and Fig. S5). Surprisingly, the E51A variant is a better substrate than WT ubiquitin (Fig. 9*B* and Fig. S5). None of the tested mutations interfere with thioester transfer of ubiquitin to the E2 or E3 (Fig. S6, *A* and *B*).

To understand the precise roles of the mutated residues in ubiquitin during E6AP-mediated isopeptide bond formation and to discriminate donor from acceptor functions, we adopted a strategy previously used for the analysis of E2–ubiquitin interactions (6, 15). This strategy involves a truncated ubiquitin variant, Ub^ΔGG^ (residues 1–74), which cannot be activated by the E1 and thus solely acts as an acceptor (Fig. 9*C*, *left*). When supplied with a mixture of Ub^ΔGG^ and full-length ubiquitin, the E6AP HECT domain assembles two distinct Ub_2_ species, which contain either two full-length ubiquitin molecules or a full-length ubiquitin molecule linked distally to a Ub^ΔGG^ variant. If one ubiquitin variant carries a His_6_-tag, these alternative reaction products can be readily separated by SDS-PAGE and provide a selective readout of mutational effects on the donor and acceptor functions during ubiquitin linkage formation. A proof-of-concept experiment is shown in Fig. 9*C* (*right*).

Using this setup, we demonstrate that residues in the hydrophobic patch (Leu-8, Ile-44, and Leu-70), as well as Gln-49, are exclusively required by the donor ubiquitin (Fig. 9, *D* and *E*). Because these residues are dispensable for thioester formation, this implies that they mediate alternative interactions of the donor during isopeptide bond formation. Mutation of Arg-42 interferes with both the donor and the acceptor ubiquitin functions (Fig. 9, *D* and *E*). In contrast, mutation of Glu-51 in the acceptor ubiquitin enhances the formation of Ub_2_ (Fig. 9, *D* and *E*).

**Figure 10.**
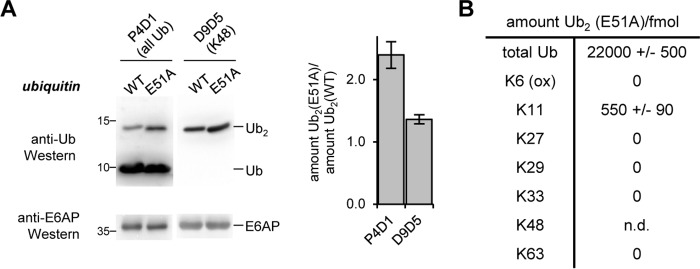
**Glu-51 of ubiquitin is a critical determinant of the Lys-48 linkage specificity of E6AP.** *A,* comparison of the activity of the E6AP HECT domain toward ubiquitin WT and the E51A variant, respectively, monitored by reducing SDS-PAGE and Western blotting. Two different anti-ubiquitin antibodies were used: P4D1 to monitor all ubiquitin modifications, and D9D5 for Lys-48 linkages. The input amount of E6AP is monitored by reducing SDS-PAGE and Western blotting against E6AP (*left*). The amounts of Ub_2_ assembled from the WT and E51A ubiquitin variants were quantified after 30 min, and the mean ratios and standard deviations from three independent experiments were plotted (*right*). *B,* amounts of Ub_2_ species linked through individual lysine residues from reactions supplied with ubiquitin E51A, measured by AQUA MS. The values reflect the means and standard deviations from three biological replicates. Note that Lys-48 linkages could not be quantified reliably in this setup, due to the E51A mutation impacting peptide ionization. The corresponding data are provided in Fig. S7. For the corresponding data on WT ubiquitin, see Fig. 8*A* and Fig. S4.

We therefore speculated that the E51A mutation may alter the recognition of the acceptor ubiquitin by E6AP and allow for the formation of linkages other than Lys-48. To test this idea, we analyzed the Ub_2_ species assembled from WT and E51A ubiquitin by Western blotting using two anti-ubiquitin antibodies, which recognize all linkage types (P4D1) and Lys-48 linkages (D9D5), respectively (Fig. 10*A*). Quantification of the relative amounts of Ub_2_ detected by these antibodies suggests that the E51A mutation in ubiquitin triggers a loss of Lys-48 specificity in E6AP and enables the formation of alternative linkage types. AQUA analysis identifies Lys-11 as the alternative linkage (Fig. 10*B* and Fig. S7). We could not accurately quantify the proportion of Lys-48 linkages formed with the E51A variant, due to the impact of the mutation on the ionization. With WT ubiquitin, however, Lys-48 linkages are exclusively formed (Fig. 8*A* and Fig. S4).

Taken together, these studies assign distinct roles to individual surface regions of ubiquitin during E6AP-mediated isopeptide bond formation. (i) The hydrophobic patch, along with Arg-42 and Gln-49, is required by the donor ubiquitin for interactions that are distinct from those formed with the C-lobe during thioester formation; these interactions may involve the N-lobe, the acceptor ubiquitin, or alternatively, an alternative interface with the C-lobe, following structural rearrangements. (ii) A hydrophilic patch adjacent to Lys-48 is utilized by the acceptor ubiquitin, with Glu-51 making critical contributions to the Lys-48 linkage specificity of E6AP.

### N-lobe of E6AP interacts with ubiquitin

**Figure 11.**
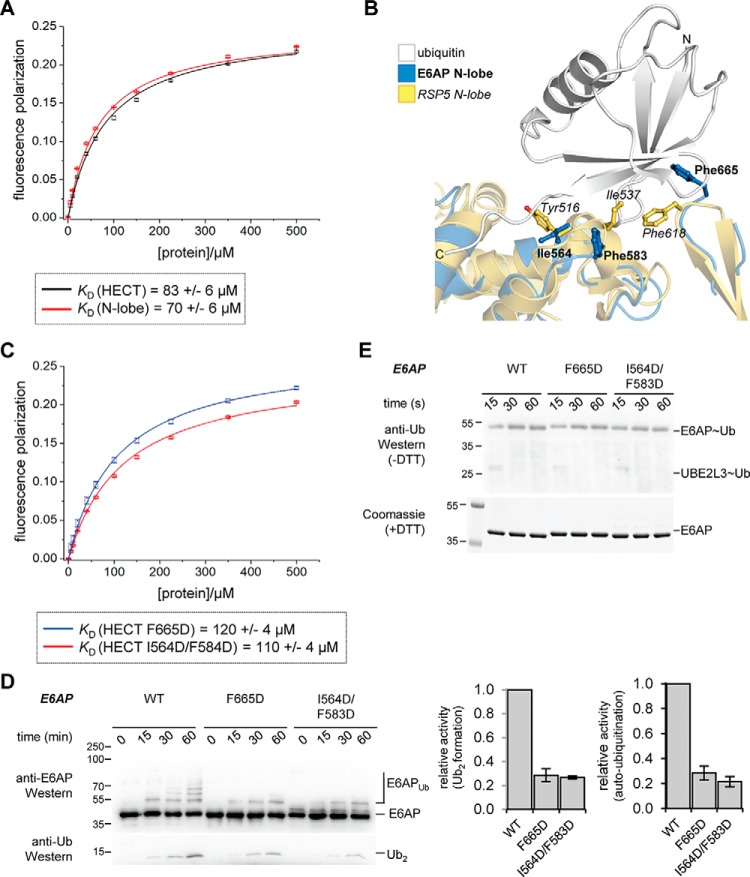
**The N-lobe of E6AP interacts with ubiquitin, and residues in the exosite of this ligase are important for isopeptide bond formation.** *A,* FP-based determination of an apparent dissociation constant, *K_D_*, for the interaction of ubiquitin with the E6AP N-lobe and the HECT domain, respectively. The mean FP signal and standard deviations from three independent experiments were fitted to a single-site binding model (*line*). *B,* structural superposition of the E6AP N-lobe (extracted from PDB code 1C4Z (30)) with the ubiquitin-bound N-lobe of RSP5 (extracted from PDB code 3OLM (36)). Protein backbones are shown as *ribbons* and mutated residues as *balls and sticks*. The C-lobes are not displayed for clarity. *C, K_D_* determination analogous to *A*, using exosite variants of the E6AP HECT domain. *D,* isopeptide bond formation assays comparing the activities of E6AP HECT domain variants. Activities are monitored at three time points, as indicated, by SDS-PAGE and Western blotting against E6AP (HECT domain auto-ubiquitination marked as *E6AP_Ub_*) and ubiquitin (di-ubiquitin reaction product marked as *Ub_2_*), respectively. Time point 0 denotes samples before ATP addition. The amounts of Ub_2_ and E6AP_Ub_ at 60 min were quantified and normalized to the E6AP input, and the means and standard deviations from three independent experiments were plotted. *E,* thioester transfer of ubiquitin from the E2 (UBE2L3) to the E6AP HECT domain, followed in single-turnover, pulse-chase assays at three time points, as indicated, and monitored by reducing SDS-PAGE and anti-ubiquitin Western blotting. The input amount of E6AP is monitored by reducing SDS-PAGE and Coomassie staining. Note that no auto-ubiquitination of the HECT domain occurs within the tested time range.

To test for interactions between ubiquitin and the N-lobe of E6AP, we performed FP experiments, analogous to our studies of the C-lobe. These experiments reveal that the isolated N-lobe indeed binds to ubiquitin. The dissociation constant, 70 ± 6 μm, is similar to the one we measured for the full HECT domain (83 ± 6 μm) (Fig. 11*A*) and is significantly tighter than that of the isolated C-lobe (400 ± 20 μm; see Fig. 1*D*).

Because the N-lobe of several NEDD4-type enzymes was shown to recognize ubiquitin via a regulatory “exosite” (18, 26, 34–38) with comparable affinities in the micromolar range (∼11 and between ∼70 and 90 μm for the HECT domains of NEDD4 and RSP5, respectively (26, 34, 36)), we investigated whether E6AP also utilizes this site. Sequence and structural alignments of E6AP with NEDD4-type enzymes show that the exosite region is moderately conserved in E6AP (Fig. S8, *A* and *B*); for example, a particular helical linker (Asn-621–Glu-629 of NEDD4) that contains a functionally critical hydrophobic residue (Leu-626, corresponding to Phe-583 of E6AP) is shortened in E6AP (Asn-625–Glu-584). We introduced nonconservative amino acid substitutions in E6AP at positions homologous to key residues in the exosite of NEDD4-type enzymes (Ile-564, Phe-583, and Phe-665 of E6AP, corresponding to Tyr-605, Leu-626, and Phe-707 of NEDD4; and Tyr-516, Ile-537, and Phe-618 of RSP5, respectively (34, 36, 37, 39)) and determined the affinities of these HECT domain variants for ubiquitin by FP (Fig. 11, *B* and *C*). Compared with the WT, ubiquitin binding to the F665D and I564D/F583D variants is only slightly weakened with *K_D_* values of 120 ± 4 and 110 ± 4 μm, respectively. In contrast, the equivalent mutations in NEDD4-type enzymes were reported to result in a drastic reduction in ubiquitin binding (*e.g.* NEDD4 F707A: *K_D_* = 340 μm; NEDD4 Y605A: *K_D_* = 87 μm (34); RSP5 F618D and I537D: *K_D_* values could not be determined (36)).

Interestingly, however, the tested mutations markedly reduce the isopeptide bond formation activity of the E6AP HECT domain (Fig. 11*D*), while leaving thioester formation intact (Fig. 11*E*). These observations mirror the effect of the exosite in certain NEDD4-type enzymes (26, 34–36, 38, 54), while recent studies using ubiquitin variant probes have indicated that the precise function of the exosite during catalysis varies across different HECT ligases (38).

Taken together, our studies uncover an important function of the exosite region in E6AP during isopeptide bond formation. Whether the newly identified interaction between the N-lobe of E6AP and ubiquitin occurs through a canonical or a distinct mode of recognition remains to be determined.

## Discussion

Ubiquitination enzymes rely on combinatorial weak interactions to achieve efficiency and specificity in ubiquitin transfer. How such interactions direct the catalysis and functional specialization of HECT ligases is incompletely understood. We addressed this question by elucidating the mechanism of ubiquitin recognition by E6AP. Our studies demonstrate that the first step in the catalytic cycle, thioester formation with ubiquitin, requires specific contacts between the C-lobe of the catalytic HECT domain and the donor ubiquitin that are formed in addition to the linkage of ubiquitin at the active site. This interface *per se* is weak and presumably dynamic, reminiscent of the functionally critical “closed” conformation of donor ubiquitin with respect to E2s in RING E3-driven catalysis (55). However, in both cases, the covalent linkage between the enzyme and the donor brings the local protein concentrations into the millimolar range (5, 6), thus rendering weak interfaces significantly populated. Additional macromolecular interactions of the binding partners with adapter proteins or in the context of higher-order complexes can also contribute to the stabilization of inherently weak interactions. For example, RING/U-box as well as ligases specific for particular ubiquitin-like modifiers (Ubls), such as SUMO and NEDD8, promote closed orientations of the donor ubiquitin/Ubl toward an E2 by simultaneously interacting with both proteins (56–65). Moreover, the human anaphase–promoting complex, a multicomponent RING ligase, holds the acceptor ubiquitin in a Lys-11–specific position toward the chain-elongating E2, UBE2S (66–68); and the Lys-63–specific E2 UBC13 relies on an associated ubiquitin E2 variant, MMS2, to orient the acceptor ubiquitin (4, 7). Multiple protein interfaces were also shown to be involved in positioning substrates for modification with Ubls (61, 62, 64). It is likely that similar combinatorial mechanisms serve to stabilize critical interfaces of ubiquitin with HECT ligases. Notably, these enzymes are characterized by extended regions flanking the C-terminal catalytic domain that recruit substrates and can impact ubiquitin recognition (39, 69–71). Furthermore, the ubiquitin-binding exosite on the N-lobe of NEDD4-type ligases was found to promote ubiquitin chain elongation in several cases, presumably by tethering a growing chain, including the acceptor ubiquitin (26, 34–36, 38, 72).

**Figure 12.**
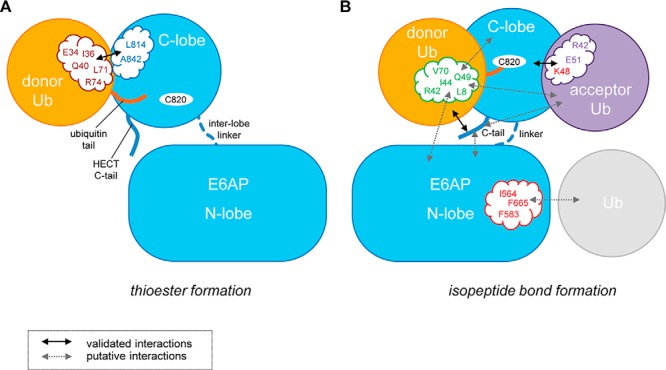
**Schematic view summarizing the identified interactions and surface patches critical for Lys-48–linked ubiquitin chain formation by the HECT domain of E6AP.** Our studies suggest that the C-lobe of E6AP utilizes canonical (NEDD4-type) contacts with the donor ubiquitin (*rose*) during thioester transfer of ubiquitin from the E2 to the E3. The C-terminal tail (*C-tail*) of E6AP is not required for this step (*A*). E6AP-mediated isopeptide bond formation between the thioester-linked donor–E6AP complex and an acceptor ubiquitin relies on surface regions that are distinct from those required during thioester transfer (*B*). The hydrophobic patch (*green*) is used by the donor ubiquitin for yet uncharacterized interactions with the N-lobe, the C-lobe, or the acceptor ubiquitin. The acceptor ubiquitin is critically dependent on a hydrophilic area (*purple*) around the acceptor residue (Lys-48), including Glu-51. The C-tail contacts the thioester-linked donor and confers linkage specificity in isopeptide bond formation, possibly by additional contacts with the N-lobe or the acceptor ubiquitin. Furthermore, we demonstrate that E6AP interacts with ubiquitin through the N-lobe. Mutations of residues in the exosite region (*red*) weaken this interaction and reduce isopeptide bond formation activity; however, they do not affect thioester formation. Whether the N-lobe–ubiquitin interaction resembles the exosite-mediated binding mode seen in NEDD4-type ligases awaits structural elucidation. It also remains unclear which functional ubiquitin moiety this interaction involves (*e.g.* a regulatory moiety or, possibly, the acceptor).

Our study reveals that the catalytic C-lobe of E6AP utilizes contacts with the donor ubiquitin for thioester formation that are equivalent to NEDD4-type ligases and HUWE1 (whose HECT domain is closely related to NEDD4-type ligases; Fig. S9*A*). Consistently, residues in the NEDD4-type donor interface are at least partially conserved in E6AP, and functionally critical residues are largely identical, despite the HECT domains being relatively distant in phylogenetic terms (Fig. S9, *A* and *B*). Taken together, these observations support the notion that thioester transfer of ubiquitin from the E2 to the E3 occurs through a structurally conserved interface between ubiquitin and the C-lobe of HECT ligases (Fig. 12*A*) (41).

During isopeptide bond formation, E6AP requires the hydrophobic patch of ubiquitin (49). Our analyses pinpoint the significance of this region to a function in the donor rather than the acceptor ubiquitin. Because the hydrophobic patch is not part of the interface required during thioester transfer, several alternative models are conceivable. For example, the hydrophobic patch may be important for interactions of the donor with the N-lobe or the acceptor ubiquitin, with the donor adopting a canonical orientation toward the C-lobe (Fig. 12*B*). Alternatively, the hydrophobic patch may mediate alternative interactions of the donor with the C-lobe, following structural rearrangements in the course of isopeptide bond formation (Fig. 12*B*). Because our NMR data point to considerable flexibility within the donor–C-lobe conjugate and the HECT domain harbors additional inter-lobe flexibility (73, 74), such rearrangements may well occur as the enzyme progresses from one sub-reaction to the next. In consequence, conformational variability in the orientations of both the donor and the acceptor ubiquitin during isopeptide bond formation may provide the basis for distinct linkage specificities of individual ligases despite a shared mode of ubiquitin recognition during thioester transfer.

To fully understand the conformational trajectory of the HECT domain of E6AP during its catalytic cycle, it will be essential to structurally visualize the acceptor ubiquitin along with the donor–E3 conjugate, a yet unresolved problem in the field of HECT ligases.

Our studies identify a ubiquitin-binding site on the N-lobe of E6AP, a feature thus far attributed primarily to NEDD4-type ligases (18, 26, 34–39). Whether ubiquitin recognition by the N-lobe of E6AP resembles a typical exosite interaction remains unclear, particularly because this site is moderately conserved, and mutation of key residues weakens ubiquitin binding to the E6AP HECT domain only slightly. However, these residues are critical for the isopeptide bond formation activity of E6AP. This latter observation mimics the function of the exosite region in certain NEDD4-type ligases, even though the specific function of this site in catalysis is enzyme-dependent (26, 34–38). To unravel the precise role of the exosite during ubiquitin chain formation by E6AP will require further investigation. On a broader scope, understanding how the exosite promotes catalysis in different HECT ligases will likely also help to clarify the directionality of ubiquitin chain elongation, for which different models have been presented (9, 26, 32, 51, 54, 75, 76), as well as the functional consequences of oligomerization, which has emerged as an important regulatory theme and has been associated with the exosite in certain cases (39, 69, 77–79).

How is the Lys-48 linkage specificity of E6AP encoded? We demonstrate that elements on both the ligase and ubiquitin are required. Consistent with previous studies identifying the C-tail of HECT ligases as a sensitive element in catalysis and critical for the Lys-63 linkage specificity of NEDD4 (18, 40, 41, 50, 51), we find that the C-tail is essential for the Lys-48 specificity of E6AP. We further show that the C-tail of E6AP interacts with the donor ubiquitin upon formation of the covalent linkage at the active site, analogous to NEDD4-type ligases and HUWE1 (41). The C-tail thus contributes to the orientation of the donor and/or the chemical environment at the active site in a manner that facilitates isopeptide bond formation (18, 41, 50). In line with this idea, the C-terminal tail of RSP5, a NEDD4-orthologue from yeast, was suggested to mediate critical interactions between the N- and C-lobes as well as donor ubiquitin during substrate modification (40) and addition of a C-tail–derived peptide to E6AP was found to inhibit its isopeptide bond formation activity noncompetitively (51). Interestingly, nonconservative amino acid substitutions and insertions in the C-tail of E6AP are associated with Angelman's syndrome (missense, G850D; deletion/insertion, A842-T844/RCS; C-terminal insertion QNKIKQKKGRKKKEKI) (80–82) and certain types of cancer (A842D and T844M) (83), likely causing E6AP to malfunction in these settings.

We uncover a surface region of ubiquitin, adjacent to Lys-48, that is important for specific acceptor ubiquitin recognition by E6AP (Fig. 12). Remarkably, the exchange of a single residue within this region, Glu-51, by alanine, reduces the Lys-48 linkage specificity of the HECT domain. It is thus tempting to speculate that the isopeptide bond formation activity of E6AP is substrate-assisted, analogous to the mechanisms employed by certain RING ligase–associated E2 enzymes (4, 6, 84). In several of those cases, it is also an acidic residue near the acceptor site that confers linkage specificity. For example, Lys-11–linked chain formation by the human anaphase–promoting complex/cyclosome–associated E2, UBE2S, critically depends on Glu-34 of the acceptor ubiquitin (6); and UBE2N/UBE2V2 relies on Glu-64 of the acceptor to form Lys-63 linkages (4). Moreover, substrate-assisted catalysis has been observed during the cleavage of ubiquitin linkages by deubiquitinating enzymes (85, 86), suggesting it presents a common mechanism during both ubiquitin chain assembly and disassembly. The intense participation of ubiquitin in catalysis—along with the extensive manner by which ubiquitin surfaces engage in protein interactions—may have contributed to the striking conservation of this versatile modifier throughout eukaryotic evolution.

## Experimental procedures

### Gene constructs

The genes encoding the HECT domain (residues 495–852; numbering according to isoform 1) and the C-lobe (residues 741–852) of E6AP were cloned into a pET-28a vector (Merck, Darmstadt, Germany) that had been modified to encode an N-terminal 3C protease–cleavable His_6_-tag. The DNA constructs encoding ubiquitin, UBA1, and UBE2L3 were previously described (6, 69). The genes encoding truncated ubiquitin variants (residues 1–74, referred to as “Ub^ΔGG^”) were subcloned into pET-28a.

### Protein expression and purification

E6AP and Ub^ΔGG^ variants were purified as His_6_-tagged proteins from *Escherichia coli* BL21 (DE3). Cells were grown at 37 °C in TB medium, supplemented with the appropriate antibiotics. At an *A*_600_ value of 0.6, protein expression was induced by the addition of 0.5 mm isopropyl 1-thio-β-d-galactopyranoside. After 12 h at 18 °C, cells were harvested by centrifugation and lysed in 50 mm Tris, pH 8.0, 100 mm NaCl, 5 mm benzamidine, 4% glycerol, 0.1% Triton X-100, 8 mm β-mercaptoethanol (β-ME), and protease inhibitor mixture (Roche Applied Science, Penzberg, Germany). After centrifugation the supernatant was applied to a HisTrap HP column (GE Healthcare, Uppsala, Sweden), washed with 50 mm Tris, pH 8.0, 400 mm NaCl, and 8 mm β-ME and eluted in the presence of 500 mm imidazole. 3C protease–mediated cleavage of the His_6_-tag was performed at 4 °C in 50 mm Tris, pH 7.5, 200 mm NaCl, and 2 mm DTT overnight. To remove the tag and the protease, a second nickel–affinity chromatography (HisTrap HP column) was performed, followed by a gel filtration (Superdex HiLoad 16/600 75 pg column, GE Healthcare) in 50 mm Tris, pH 7.5, 75 mm NaCl, and 2 mm DTT.

Full-length ubiquitin variants, UBA1 and UBE2L3, were prepared as described previously (6, 69). ^15^N-Enriched proteins were expressed in M9-medium supplemented with ^15^N-enriched ammonium chloride (Sigma) and purified as described above.

### Preparation of a stable E6AP C-lobe–ubiquitin conjugate

To obtain a stable mimic of the E6AP C-lobe–ubiquitin complex, we replaced the native thioester linkage by a disulfide bond. To this end, we activated a G76C variant of ubiquitin by 5,5′-dithiobisnitrobenzoic acid (DTNB), followed by a disulfide exchange reaction with the single cysteine-containing C-lobe, as described in previous studies on E2s (87). In short, the required purified proteins were buffer-exchanged into 75 mm sodium phosphate, pH 7.5, and ubiquitin-activated by incubation with a 9-fold molar excess of DTNB at room temperature for 30 min. Excess DTNB was then removed (HiPrep 26/10 desalting column, GE Healthcare), and the DTNB-modified ubiquitin was incubated with a sub-stoichiometric amount of the E6AP C-lobe at room temperature for 30 min. After buffer exchange into 25 mm Tris, pH 6.5, the disulfide-linked conjugate was purified by cation-exchange chromatography (Mono S 4.6/100 PE, GE Healthcare), using a gradient of 0–500 mm NaCl and size-exclusion chromatography (SD 75 16/600, GE Healthcare) in 75 mm sodium phosphate, pH 7.5, and 1 mm EDTA.

### NMR spectroscopy

Data were recorded at 25 °C, using a Bruker DRX 700 MHz spectrometer, equipped with a ^1^H/^15^N/^13^C cryo-probe. Backbone resonance assignments for the E6AP C-lobe and ubiquitin were taken from the Biological Magnetic Resonance Bank (accession numbers 5013 (88) and 17437 (6)). Chemical shift mapping was performed based on titration experiments by mixing two stock solutions containing 200 μm ^15^N-enriched E6AP C-lobe in 75 mm sodium phosphate, pH 7.4, 10% D_2_O, 5 mm DTT, 2 mm TCEP and either no excess or a maximum excess of ubiquitin at the desired ratios. Phase-sensitive, gradient-enhanced ^1^H-^15^N HSQC (89) and BEST-TROSY (90) spectra were recorded for ^15^N-enriched ubiquitin and E6AP, respectively. Weighted combined chemical shift perturbations, Δδ(^1^H^15^N), were calculated according to Equation 1.

(Eq. 1) Δδ(H1N15)=(δ(H1)−δ(H1)0)2+0.04·(δ(N15)−δ(N15)0)2

In the case of the E6AP C-lobe, missing values are due to prolines (residues 793, 809, 815, and 827) or missing assignments (residues 820, 764, and 795). In the case of ubiquitin, gaps are due to prolines (residues 19, 37, and 38).

### Activity assays

To monitor the formation of unattached ubiquitin chains (readout by virtue of di-ubiquitin) and E6AP auto-ubiquitination, 0.2 μm UBA1, 2 μm UBE2L3, and 200 μm ubiquitin WT or variants were preincubated in 50 mm Tris, pH 7.0, 75 mm NaCl, 10 mm MgCl_2_, and 2 mm ATP at 30 °C for 30 min; then 2 μm E6AP HECT domain WT or variants were added for the indicated reaction times.

To test the effects of mutations in the donor and acceptor ubiquitin, full-length ubiquitin and His_6_-Ub^ΔGG^ variants were mixed at 100 μm concentration (each) with 0.2 μm UBA1, 2 μm UBE2L3, and 2 μm E6AP HECT domain in 50 mm Tris, pH 7.0, 75 mm NaCl, 10 mm MgCl_2_, and 2 mm ATP at 30 °C for 60 min.

To monitor ubiquitin thioester transfer from UBE2L3 to E6AP, we performed single-turnover, pulse-chase assays. Here, 1 μm UBA1, 10 μm UBE2L3, and 200 μm ubiquitin WT or variants thereof were incubated in 50 mm Tris, pH 7.0, 75 mm NaCl, 0.1 mm DTT, 10 mm MgCl_2_, and 2 mm ATP at 30 °C for 30 min; the reactions were quenched by 4-fold dilution in the same buffer, but including 25 mm EDTA and stored on ice for 30 min. The UBE2L3–ubiquitin conjugate was then incubated with 5 μm E6AP for the indicated reaction times.

Thioester transfer of ubiquitin from the E1 to the E2 was monitored by incubating 1 μm UBA1, 10 μm UBE2L3, and 200 μm ubiquitin WT or variants thereof in 50 mm Tris, pH 7.0, 75 mm NaCl, 0.1 mm DTT, 10 mm MgCl_2_, and 2 mm ATP at 30 °C for the indicated reaction times.

All reactions were quenched by addition of reducing and nonreducing SDS loading dye, respectively, as specified and analyzed by SDS-PAGE, followed by either Coomassie staining, silver staining or Western blotting with the following antibodies: anti-UBE3A (Cell Signaling Technology, Danvers, MA; RRID: AB_10971637); anti-UBE2L3 (Cell Signaling Technology; RRID: AB_10829170); anti-ubiquitin P4D1 SC-8017 (Santa Cruz Biotechnology, Dallas, TX; RRID: AB_2315523); or anti-Lys-48 linkage (Boston Biochem, Cambridge, MA; RRID: AB_2490534).

Where appropriate, reaction input and products were quantified with ImageJ (RRID: SCR_003070) (91). The means and standard deviations from at least three independent experiments were plotted.

### Fluorescence polarization

FP measurements were performed analogously to previous studies (34). In short, the thiol-reactive fluorescent probe BODIPY® TMR C5-maleimide (ThermoFisher Scientific, Waltham, MA) was dissolved in DMSO and incubated at a 10-fold molar excess with the ubiquitin G76C variant in reaction buffer (20 mm HEPES, pH 7.2, 200 mm NaCl, 5% glycerol, and 1 mm TCEP) at 4 °C overnight. The separation of the unreacted fluorophore was achieved by three rounds of dialysis, followed by a size-exclusion chromatography (Superdex 75 16/600 column, GE Healthcare) in reaction buffer.

FP measurements were carried out at room temperature in 20 mm HEPES, pH 7.2, 200 mm NaCl, 1 mm TCEP, and 0.01% Triton X-100 in 96-well flat-bottom microplates (Greiner Bio-One, Frickenhausen, Germany), using a Clariostar microplate reader (BMG Labtech, Ortenberg, Germany) at 540 nm excitation and 590 nm emission wavelengths. The peptide concentration was 50 nm, whereas that of the E6AP C-lobe ranged from 0 to 500 μm. FP reads from three independent experiments were averaged and fitted to a single-site binding model, as described previously (69).

### Trypsin digestion and AQUA high–resolution and accurate mass MS analysis

Reactions were separated by SDS-PAGE (12% NuPAGE BisTris gels; ThermoFisher Scientific), and in-gel trypsin digestion was performed as described previously (92). Extracted peptides were frozen and dried to completion in a speed-vac. Samples were then supplemented with ubiquitin AQUA peptides (Cell Signaling Technology) and oxidized with 0.15% TFA, 0.3% hydrogen peroxide at 4 °C for 12 h.

Peptides were separated using an Easy nLC 1000 UHPLC (ThermoFisher Scientific) equipped with a homemade ProntoSIL C18 (75 μm × 15 cm) column. A linear gradient of 0–50% of solvent B over solvent A for 30 min, 50–95% of solvent B for 3 min, and 95% hold of solvent B for 7 min (solvent A: 0.1% formic acid in water; solvent B: 0.1% formic acid in acetonitrile was applied with a flow rate of 300 nl/min). For HR/AM AQUA analysis the UHPLC system was coupled with an Orbitrap Fusion^TM^ Tribid^TM^ mass spectrometer (ThermoFisher Scientific). The resolving power of the mass analyzer was set to 60,000; spectra were recorded over a range of 300–1500 *m*/*z*. For data-dependent MS/MS, the top four most intense ions with charge states of 2–5 were selected using an isolation window of 2 *m*/*z*. Fragmentation was achieved by collision-induced dissociation at 35% nominal energy with product ion detection in the linear ion trap. Ion chromatograms were extracted for each ubiquitin peptide of interest with a window of 5 ppm. Chromatograms were smoothed using the Boxcar algorithm with a 7-point window. Integration was performed using default parameters with manual adjustment, as appropriate. Results were normalized to the total amount of ubiquitin for each linkage type detected and represented from three replicates.

### pK*_a_* determination

p*K_a_* values for the thiol group of the catalytic cysteine (Cys-820) in E6AP C-lobe variants (WT and Δ4) were derived from the reaction kinetics of 100 μm DTNB and 14.4 μm protein at 10 °C, as monitored by UV absorbance (λ = 412 nm) using an SFM-3000S stopped-flow spectrometer (Bio-Logic, Seyssinet-Pariset, France). Proteins were in 100 mm NaCl, 1 mm EDTA, and one of the following buffers covering a pH range from 5 to 10.5: 20 mm sodium acetate; 20 mm sodium phosphate; 20 mm Tris; or 20 mm CAPSO. Each reaction was measured at least five times under identical conditions, and the data were averaged and fitted with single exponential functions, using the BioKine (Bio-Logic) software. Three independent experimental replicates were performed, and the resulting rate constants were averaged, plotted against the pH value, and fitted with OriginPro 2017 (OriginLab, Northampton, MA) to a two-state model, as given by Equation 2,

(Eq. 2) $$k_{\text{app}}=k_{\text{SH}}+\frac{k_{\text{S}-}-k_{\text{SH}}}{1+10^{\text{p}K_{a}-\text{pH}}}$$

where *k*_app_ is the experimentally derived apparent rate constant; and *k*_SH_ and *k*_S−_ are the rate constants of the reaction of fully protonated and fully deprotonated cysteines, respectively (93).

### Structure representation

All structural figures were generated with the PyMOL Molecular Graphics System, Version 2.0 Schrödinger, LLC.

## Author contributions

L. K. R., B. S., K. K. D., M.-A. L., E. R. S., and S. L. formal analysis; L. K. R., K. K. D., E. R. S., and S. L. funding acquisition; L. K. R., B. S., K. K. D., M.-A. L., E. R. S., and S. L. validation; L. K. R., B. S., K. K. D., M.-A. L., and S. L. investigation; L. K. R., B. S., K. K. D., M.-A. L., E. R. S., and S. L. visualization; L. K. R., K. K. D., and S. L. writing-original draft; L. K. R., B. S., E. R. S., and S. L. writing-review and editing; E. R. S. and S. L. supervision; S. L. conceptualization; S. L. methodology; S. L. project administration.

## Supplementary Material

Supporting Information

## Footnotes

5 The abbreviations used are:

E1: ubiquitin-activating enzyme
E2: ubiquitin-conjugating enzyme
E3: ubiquitin ligase
FP: fluorescence polarization
BisTris: 2-[bis(2-hydroxyethyl)amino]-2-(hydroxymethyl)propane-1,3-diol
β-ME: β-mercaptoethanol
DTNB: 5,5′-dithiobisnitrobenzoic acid
TCEP: tris(2-carboxyethyl)phosphine
CAPSO: 3-(cyclohexylamino)-2-hydroxy-1-propanesulfonic acid
AQUA: absolute quantification
Ub: ubiquitin
Ubl: ubiquitin-like modifier
PDB: Protein Data Bank.
