# Supplementary material for: Analysis of ubiquitin recognition by the HECT ligase E6AP provides insight into its linkage specificity
Source: J Biol Chem. 2019 Feb 8;294(15):6113–29. doi: 10.1074/jbc.RA118.007014 (PMC6463701; doi:10.1074/jbc.RA118.007014)
Supplement: Supporting Information [file supp_294_15_6113__index.html]

Analysis of ubiquitin recognition by the HECT ligase E6AP provides insight into its linkage specificity — Ubiquitin recognition by E6AP — Analysis of ubiquitin recognition by the HECT ligase E6AP provides insight into its linkage specificity — Ubiquitin recognition by E6AP — Supporting Information 

# Analysis of ubiquitin recognition by the HECT ligase E6AP provides insight into its linkage specificity

## Supporting Information

- Supporting Information (to be published online) - title page, supplementary figures S1-S9, supplementary references
