# Supplementary material for: Analysis of ubiquitin recognition by the HECT ligase E6AP provides insight into its linkage specificity
Source: J Biol Chem. 2019 Feb 8;294(15):6113–29. doi: 10.1074/jbc.RA118.007014 (PMC6463701; doi:10.1074/jbc.RA118.007014)
Supplement: Supporting Information [file supp_RA118.007014_142389_1_supp_275510_plyfw6.pdf]

Analysis of ubiquitin recognition by the HECT ligase E6AP provides insight into its linkage specificity

Lena K. Ries, Bodo Sander, Kirandeep K. Deol, Marie-Annick Letzelter, Eric R. Strieter, and Sonja Lorenz

- **S1:** The NMR-derived mutations in ubiquitin do not disrupt thioester formation with UBE2L3.
- **S2:** Addition of ubiquitin to an E6AP-ubiquitin conjugate does not reveal an additional ubiquitin binding site on the C-lobe.
- **S3:** The C-tail of E6AP does not impact thioester formation of the HECT domain with ubiquitin nor the reactivity of the catalytic cysteine in the context of the isolated C-lobe.
- **S4:** AQUA mass spectrometric analysis of Ub_2-linkage types formed by different E6AP HECT domain variants.
- **S5:** The hydrophobic patch and a hydrophilic region adjacent to Lys48 of ubiquitin are important for E6AP activity.
- **S6:** Mutations in the hydrophobic patch or in a hydrophilic region adjacent to Lys48 of ubiquitin do not disrupt thioester transfer of ubiquitin to UBE2L3 nor to E6AP.
- **S7:** AQUA mass spectrometric analysis of Ub_2-linkage types formed by the E6AP HECT domain with E51A ubiquitin.
- **S8:** Residues in the ubiquitin-binding exosite of NEDD4-type ligases are partially conserved in E6AP.
- **S9:** Phylogenetic analyses of human HECT domains

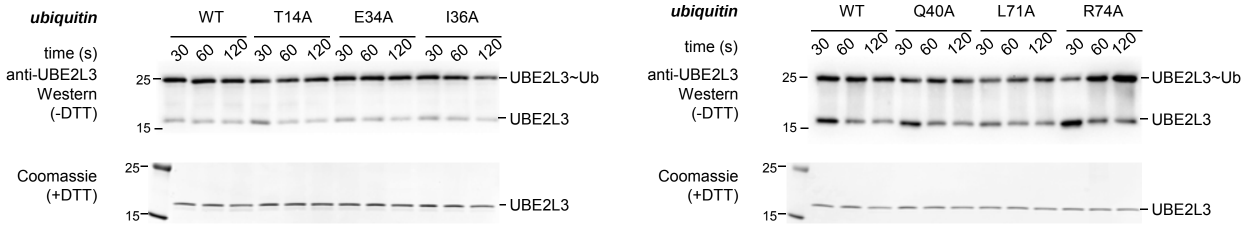

Figure S1. The NMR-derived mutations in ubiquitin do not disrupt thioester formation with UBE2L3. Thioester transfer of ubiquitin variants from the E1 (UBA1) to the E2 (UBE2L3), followed in single-turnover, pulse-chase assays at 3 time points, as indicated, and monitored by non-reducing SDS-PAGE and anti-UBE2L3 Western blotting. The input amount of E2 ('UBE2L3') is monitored by reducing SDS-PAGE and Coomassie staining.

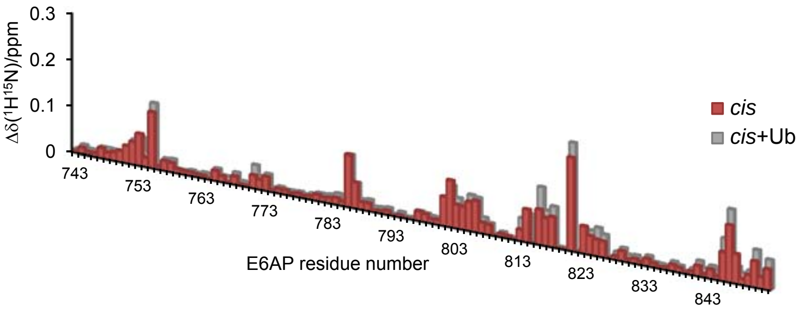

Figure S2. Addition of ubiquitin to an E6AP-ubiquitin conjugate does not reveal an additional ubiquitin binding site on the C-lobe.

Weighted, combined chemical shift perturbations, $\Delta\delta(^{1}H^{15}N)$, of E6AP C-lobe resonances in the context of a covalent conjugate (*in cis*, see Figure 5) in the absence or presence of additional ubiquitin (at 15-fold molar excess).

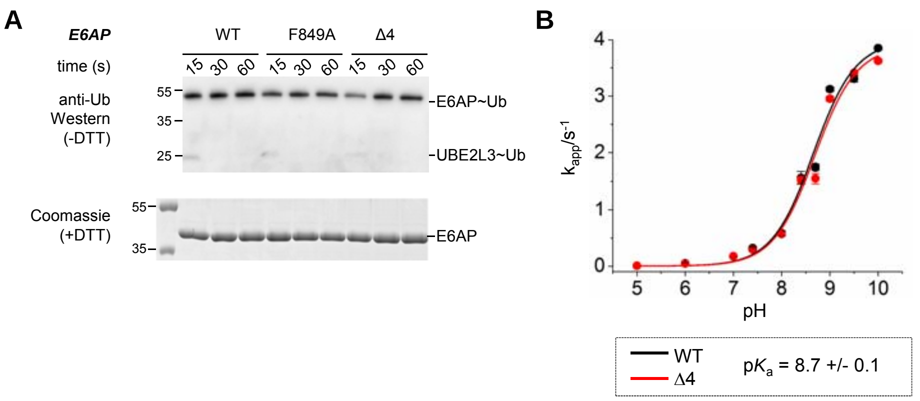

Figure S3. The C-tail of E6AP does not impact thioester formation of the HECT domain with ubiquitin nor the reactivity of the catalytic cysteine in the context of the isolated C-lobe.

(A) Thioester transfer of ubiquitin from the E2 (UBE2L3) to the E6AP HECT domain, followed in single-turnover, pulse-chase assays at 3 time points, as indicated, and monitored by non-reducing SDS-PAGE and anti-ubiquitin Western blotting. The thioester-linked HECT domain-ubiquitin conjugate ('E6AP~Ub') and, in some cases, the thioester-linked E2-ubiquitin precursor ('UBE2L3~Ub') are visible. The input amount of the HECT domain ('E6AP') is monitored by reducing SDS-PAGE and Coomassie staining. (B) Reaction rates of the catalytic cysteine in E6AP C-lobe variants towards DTNB (5,5'-dithio-bis-nitrobenzoic acid) at different pH-values were monitored by stopped-flow absorbance measurements. The means and standard deviations from 3 independent experiments (standard deviations are mostly too small to be visible) were fitted to a 2-state model (lines).

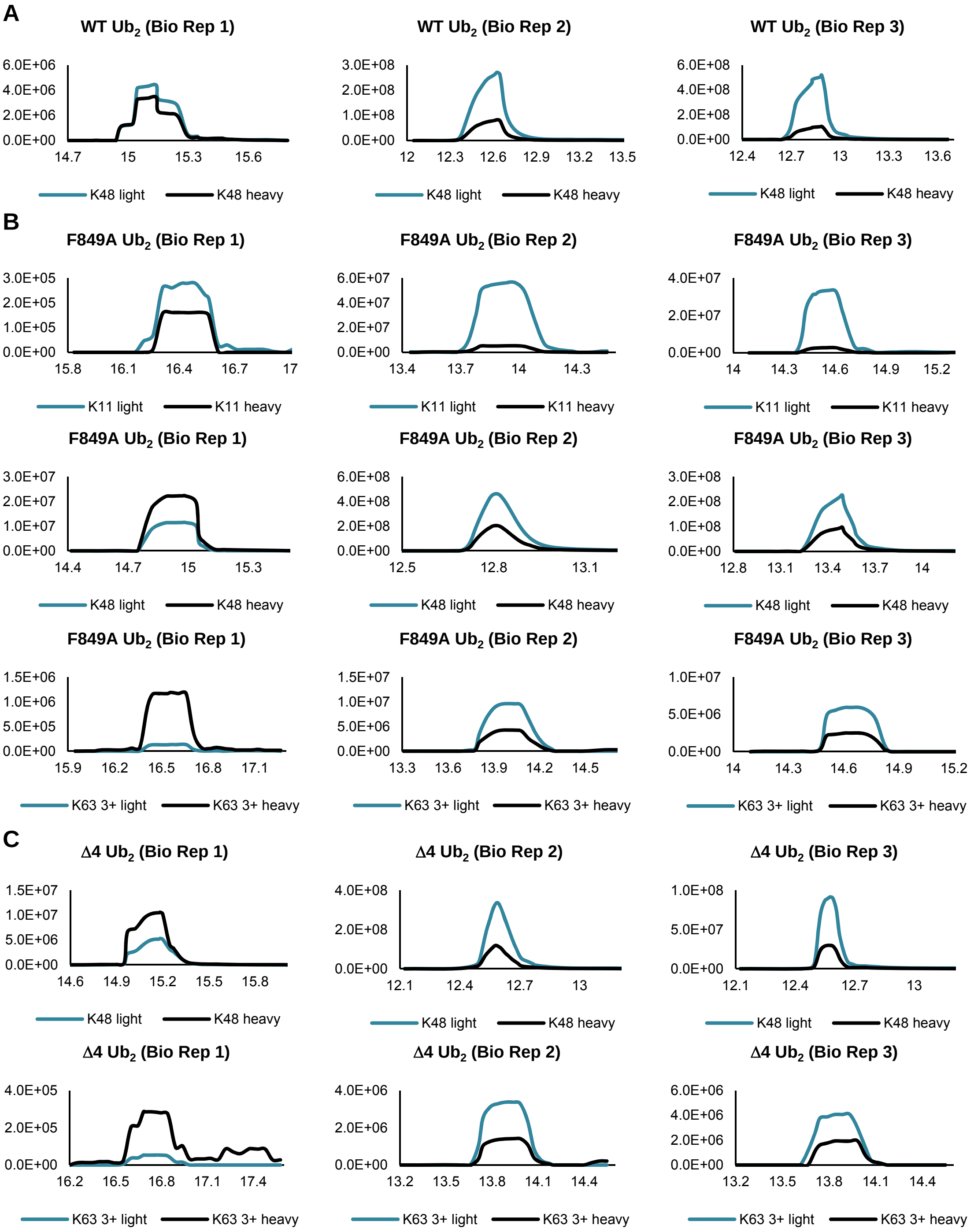

Figure S4. AQUA mass spectrometric analysis of Ub_2-linkage types formed by different E6AP HECT domain variants.

The di-ubiquitin (Ub_2) reaction products formed by the E6AP HECT domain WT (A), F849A (B), and Δ4 (C), respectively, were analyzed; extracted ion chromatograms are shown. Pairs of the co-eluting light and heavy peaks are displayed on the same relative abundance scale (y-axis) as a function of the retention time (x-axis, in minutes).

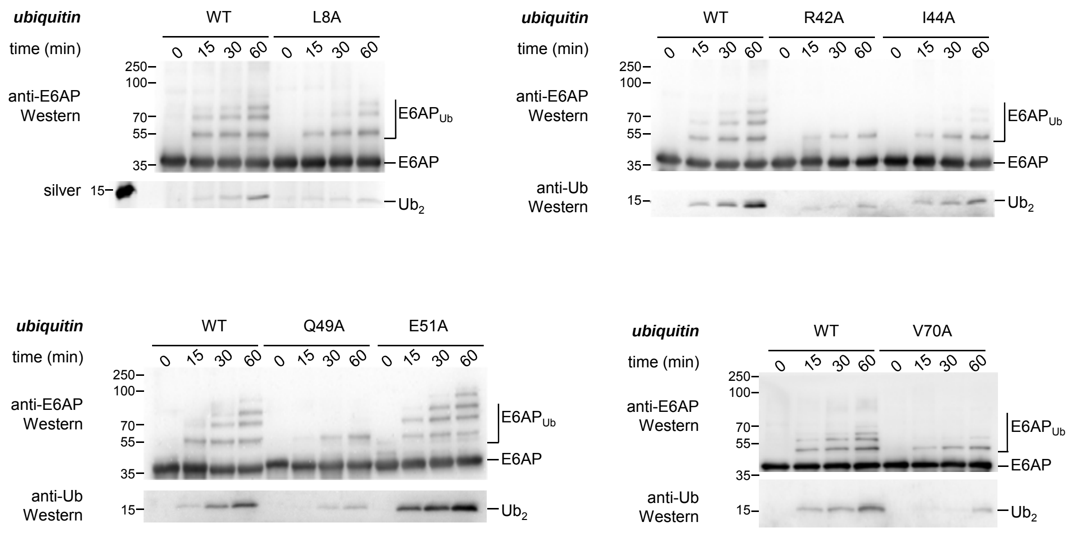

Figure S5. The hydrophobic patch and a hydrophilic region adjacent to Lys48 of ubiquitin are important for E6AP activity.
Isopeptide bond formation assays comparing the activities of the E6AP HECT domain towards different ubiquitin variants. Activities are monitored at 3 time points, as indicated, by reducing SDS-PAGE and Western blotting against E6AP (HECT domain auto-ubiquitination marked as ‘E6AP$_{Ub}$’) and ubiquitin (di-ubiquitin reaction product marked as ‘Ub$_2$’), respectively. For the L8A variant, silver staining was used in lieu of anti-ubiquitin Western blotting, since this variant is not detected well by the ubiquitin antibody (P4D1) used here. Time point zero denotes samples before ATP addition. For the corresponding quantifications, see Figure 9B.

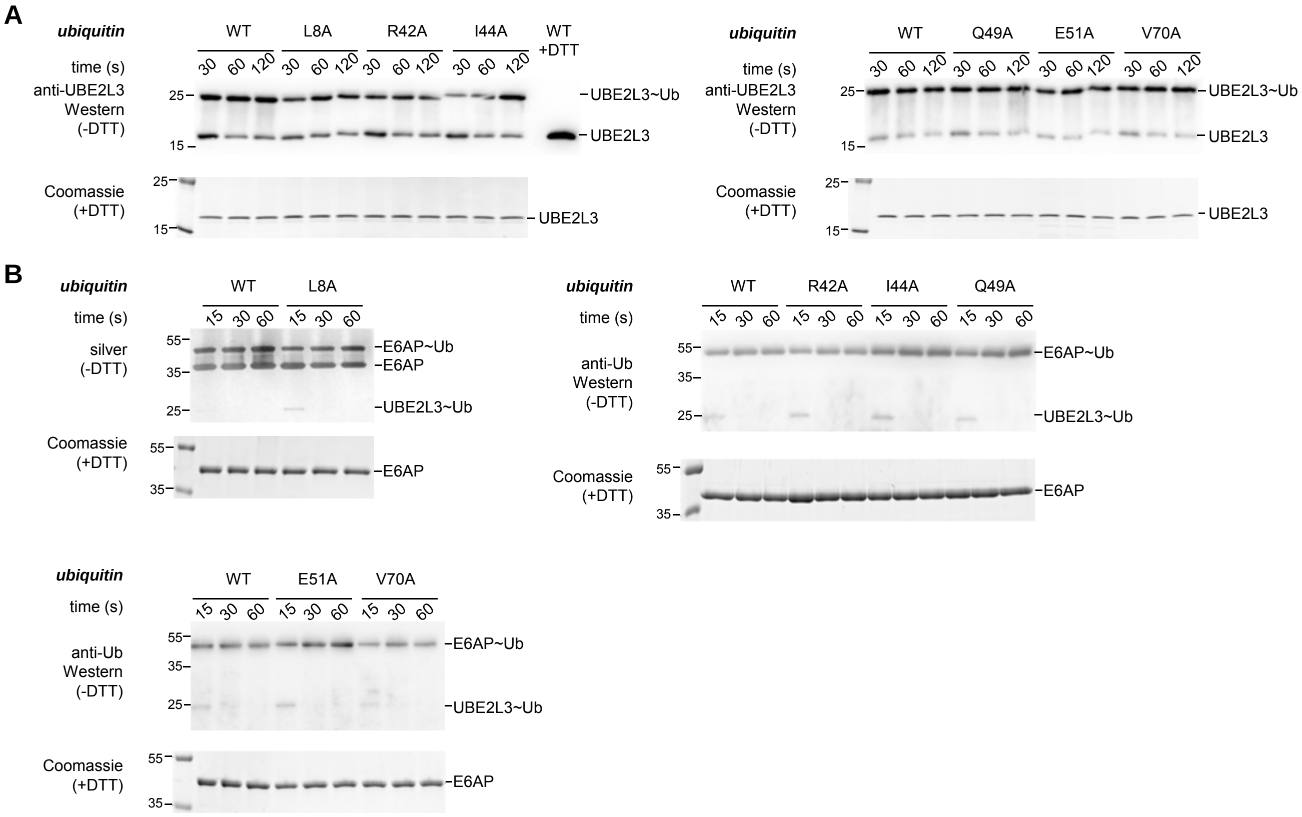

Figure S6. Mutations in the hydrophobic patch or in a hydrophilic region adjacent to Lys48 of ubiquitin do not disrupt thioester transfer of ubiquitin to UBE2L3 nor to E6AP.

(A) Thioester transfer of ubiquitin variants from the E1 (UBA1) to the E2 (UBE2L3), followed in single-turnover, pulse-chase assays at 3 time points, as indicated, and monitored by non-reducing SDS-PAGE and anti-UBE2L3 Western blotting. The input amount of E2 (‘UBE2L3’) is monitored by reducing SDS-PAGE and Coomassie staining. (B) Thioester transfer of ubiquitin variants from the E2 (UBE2L3) to the E6AP HECT domain, followed in single-turnover, pulse-chase assays at 3 time points, as indicated, and monitored by non-reducing SDS-PAGE and anti-ubiquitin Western blotting. For the L8A variant, silver staining was used in lieu of anti-ubiquitin Western blotting, since this variant is not detected well by the ubiquitin antibody (P4D1) used here. The thioester-linked HECT domain-ubiquitin conjugate (‘E6AP~Ub’) and, in some cases, the thioester-linked E2-ubiquitin precursor (‘UBE2L3~Ub’) are visible. The input amount of HECT domain (‘E6AP’) is monitored by reducing SDS-PAGE and Coomassie staining.

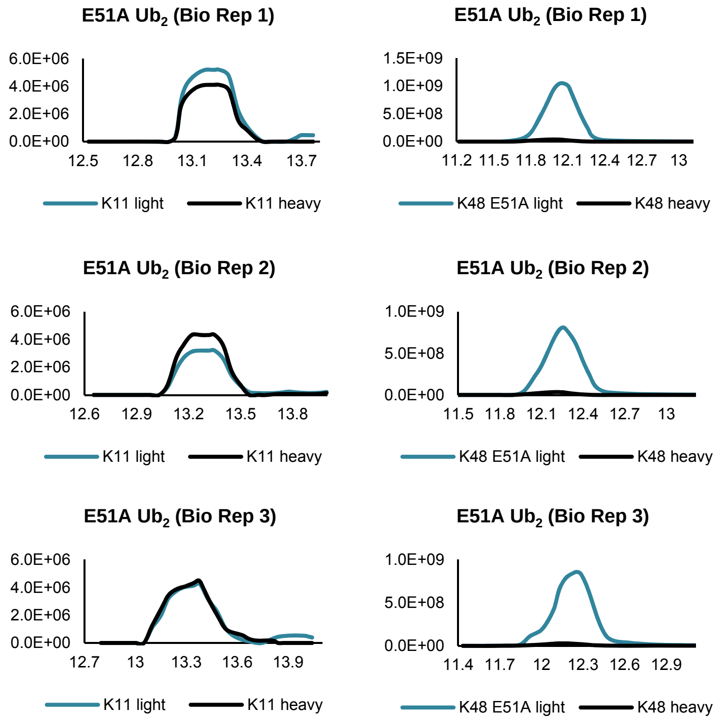

Figure S7. AQUA mass spectrometric analysis of Ub_2-linkage types formed by the E6AP HECT domain with E51A ubiquitin.

Extracted ion chromatograms are shown; pairs of the co-eluting light and heavy peaks are displayed on the same relative abundance scale (y-axis) as a function of the retention time (x-axis, in minutes).

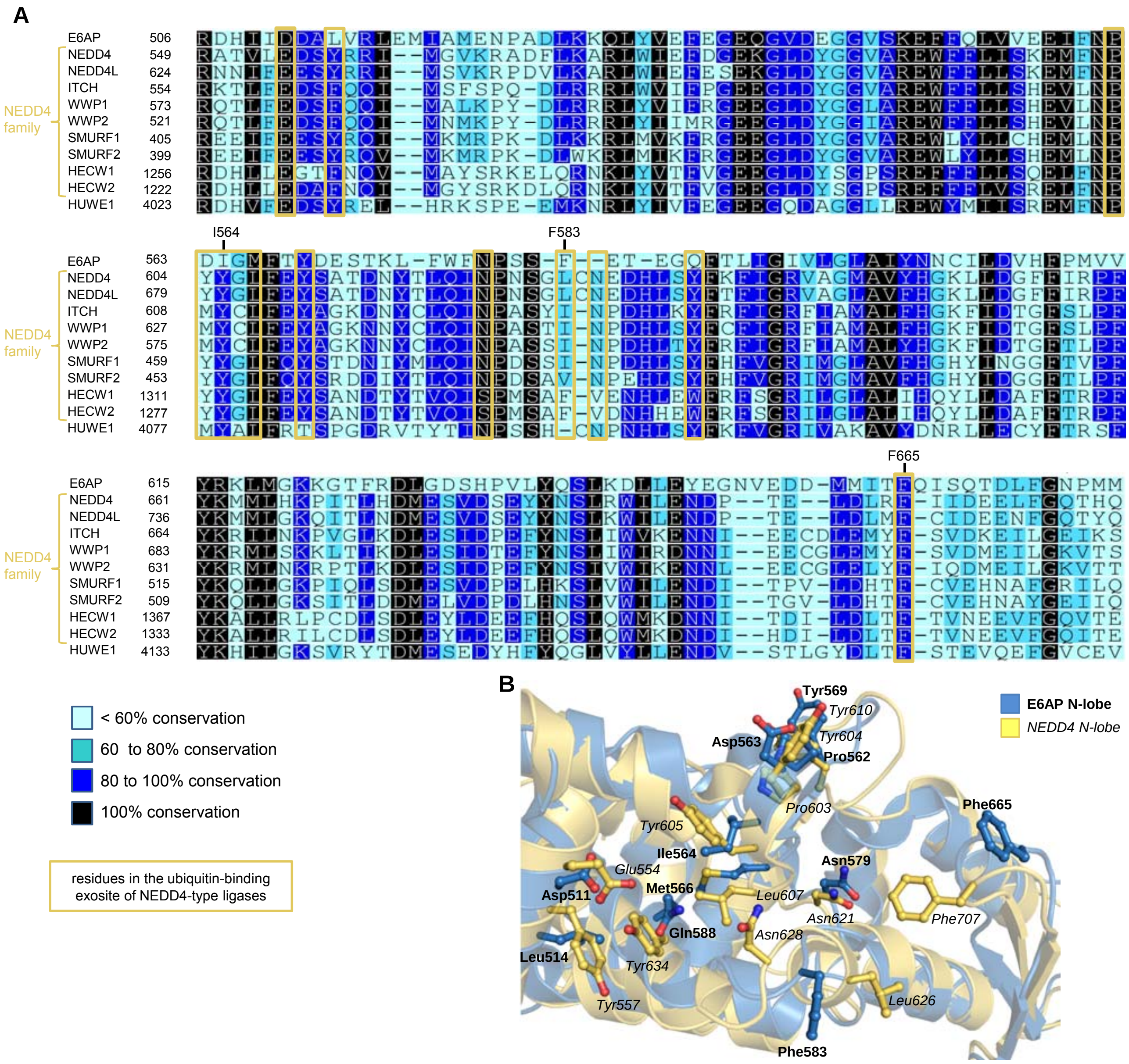

Figure S8. Residues in the ubiquitin-binding exosite of NEDD4-type ligases are partially conserved in E6AP.

(A) Amino acid sequence alignment for the N-lobe of E6AP, the members of the NEDD4 subfamily (NEDD4 isoform 4, all others isoform 1), and HUWE1, as output by Clustal Omega (RRID:SCR_001591; http://www.ebi.ac.uk/Tools/msa/clustalo/) (1), illustrated with Geneious Basic (RRID:SCR_010519) (2). Residues in the exosite were defined based on the crystal structure of the ubiquitin-bound HECT domain of NEDD4 (PDB ID: 4BBN (3)) and a minimum surface burial upon complex formation of at least 50%, determined with the PISA server (http://www.ebi.ac.uk/ pdbe/prot_int/pistart.html (4)). Residue numbers provided above the alignment refer to E6AP. (B) Structural superposition of the N-lobe of E6AP (extracted from PDB ID: 1C4Z (5)) and NEDD4 (extracted from PDB ID: 2XBB (6)); the C-lobes are not displayed for clarity. The proteins are shown in ribbon representation; the side chains of residues in the exosite (see (A)) are displayed in ball-and-stick mode (residue labels for E6AP in bolt and NEDD4 in italic).

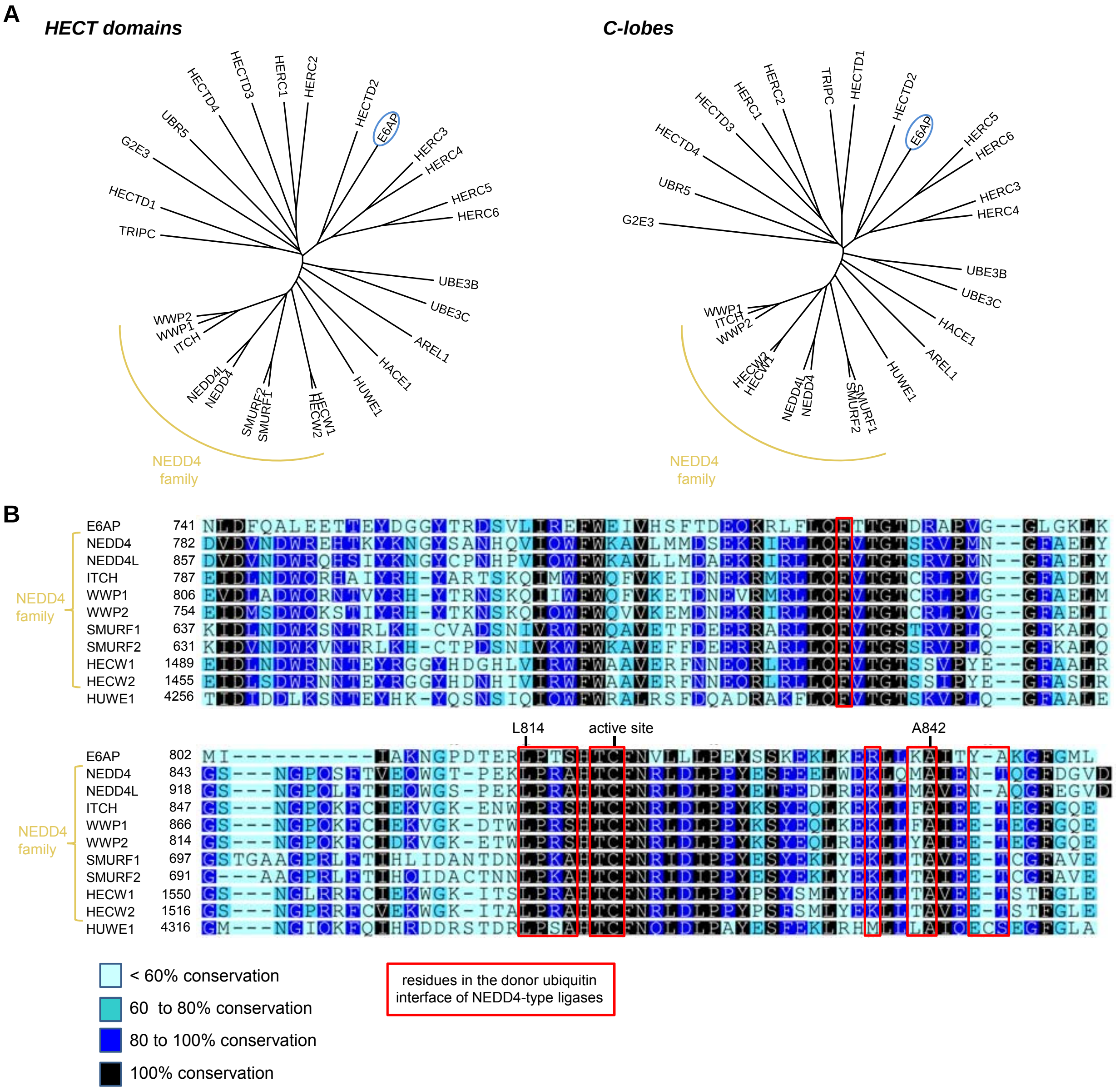

Figure S9. Phylogenetic analyses of human HECT domains

(A) Phylogenetic trees for all human HECT domains (left) and C-lobes (right), generated with Clustal Omega (1) and the Interactive Tree of Life (iTOL) v3 server (7)**.** (B) Amino acid sequence alignment for the C-lobes of E6AP, the members of the NEDD4 subfamily, and HUWE1 (NEDD4 isoform 4, others isoform 1), as output by Clustal Omega (1), illustrated using Geneious Basic (2). The catalytic cysteine and residues in the NEDD4-type interface with the donor ubiquitin are boxed. Interfacing residues were defined based on the crystal structure of the NEDD4 HECT domain-donor ubiquitin complex (PDB ID: 4BBN) (3)) and a surface burial upon complex formation of at least 50%, as determined by the PISA server (4). Residue numbers provided above the alignment refer to E6AP.

References

1. Sievers, F., Wilm, A., Dineen, D., Gibson, T. J., Karplus, K., Li, W., Lopez, R., McWilliam, H., Remmert, M., Söding, J., Thompson, J. D., and Higgins, D. G. (2011) Fast, scalable generation of high-quality protein multiple sequence alignments using Clustal Omega. *Mol Syst Biol*. **7**, 539
2. Kearse, M., Moir, R., Wilson, A., Stones-Havas, S., Cheung, M., Sturrock, S., Buxton, S., Cooper, A., Markowitz, S., Duran, C., Thierer, T., Ashton, B., Meintjes, P., and Drummond, A. (2012) Geneious Basic: an integrated and extendable desktop software platform for the organization and analysis of sequence data. *Bioinformatics*. **28**, 1647–1649
3. Maspero, E., Valentini, E., Mari, S., Cecatiello, V., Soffientini, P., Pasqualato, S., and Polo, S. (2013) Structure of a ubiquitin-loaded HECT ligase reveals the molecular basis for catalytic priming. *Nat Struct Mol Biol*. **20**, 696–701
4. Krissinel, E., and Henrick, K. (2007) Inference of macromolecular assemblies from crystalline state. *J Mol Biol*. **372**, 774–797
5. Huang, L., Kinnucan, E., Wang, G., Beaudenon, S., Howley, P. M., Huibregtse, J. M., and Pavletich, N. P. (1999) Structure of an E6AP-UbcH7 complex: insights into ubiquitination by the E2-E3 enzyme cascade. *Science*. **286**, 1321–1326
6. Maspero, E., Mari, S., Valentini, E., Musacchio, A., Fish, A., Pasqualato, S., and Polo, S. (2011) Structure of the HECT:ubiquitin complex and its role in ubiquitin chain elongation. *EMBO Rep*. **12**, 342–349
7. Letunic, I., and Bork, P. (2016) Interactive tree of life (iTOL) v3: an online tool for the display and annotation of phylogenetic and other trees. *Nucleic Acids Research*. **44**, W242–5
